# Exploring Metabolic and Gut Microbiome Responses to Paraquat Administration in Male Wistar Rats: Implications for Oxidative Stress

**DOI:** 10.3390/antiox13010067

**Published:** 2024-01-01

**Authors:** Julia Hernandez-Baixauli, Gertruda Chomiciute, Harry Tracey, Ignasi Mora, Antonio J. Cortés-Espinar, Javier Ávila-Román, Nerea Abasolo, Hector Palacios-Jordan, Elisabet Foguet-Romero, David Suñol, Mar Galofré, Juan María Alcaide-Hidalgo, Laura Baselga-Escudero, Josep M. del Bas, Miquel Mulero

**Affiliations:** 1 Eurecat, Centre Tecnològic de Catalunya, Unitat de Nutrició i Salut, 43204 Reus, Spain; julia.hernandez@vhir.org (J.H.-B.); gertruda.chomiciute@eurecat.org (G.C.); harry.tracey@eurecat.org (H.T.); juanmaria.alcaide@eurecat.org (J.M.A.-H.); lbaselga@xtec.cat (L.B.-E.); 2 Laboratory of Metabolism and Obesity, Vall d’Hebron-Institut de Recerca, Universitat Autònoma de Barcelona, 08035 Barcelona, Spain; 3 Department of Medical Sciences, School of Medicine, University of Girona, 17004 Girona, Spain; 4 School of Science, RMIT University, Bundoora, VIC 3000, Australia; 5 Brudy Technology S.L., 08006 Barcelona, Spain; cultivos1@brudylab.com; 6 Nutrigenomics Research Group, Department of Biochemistry and Biotechnology, Universitat Rovira i Virgili, 43007 Tarragona, Spain; antoniojesus.cortes@urv.cat; 7 Molecular and Applied Pharmacology Group (FARMOLAP), Department of Pharmacology, Universidad de Sevilla, 41012 Sevilla, Spain; javieravila@us.es; 8 Eurecat, Centre Tecnològic de Catalunya, Centre for Omic Sciences (COS), Joint Unit Universitat Rovira i Virgili-EURECAT, 43204 Reus, Spain; nerea.abasolo@eurecat.org (N.A.); hector.palacios@eurecat.org (H.P.-J.); elisabet.foguet@eurecat.org (E.F.-R.); 9 Eurecat, Centre Tecnològic de Catalunya, Digital Health, 08005 Barcelona, Spain; david.sunol@eurecat.org (D.S.); mar.galofre@eurecat.org (M.G.); 10 Eurecat, Centre Tecnològic de Catalunya, Àrea Biotecnologia, 43204 Reus, Spain

**Keywords:** paraquat, oxidative stress, metabolism, gut microbiome, 3-hydroxibutiric acid, sphingomyelins, lysophospholipids, *Akkermansia muciniphila*, *Escherichia coli*

## Abstract

In this study, we examined the metabolic and gut microbiome responses to paraquat (PQ) in male Wistar rats, focusing on oxidative stress effects. Rats received a single intraperitoneal injection of PQ at 15 and 30 mg/kg, and various oxidative stress parameters (i.e., MDA, SOD, ROS, 8-isoprostanes) were assessed after three days. To explore the omic profile, GC-qTOF and UHPLC-qTOF were performed to assess the plasma metabolome; ^1^H-NMR was used to assess the urine metabolome; and shotgun metagenomics sequencing was performed to study the gut microbiome. Our results revealed reductions in body weight and tissue changes, particularly in the liver, were observed, suggesting a systemic effect of PQ. Elevated lipid peroxidation and reactive oxygen species levels in the liver and plasma indicated the induction of oxidative stress. Metabolic profiling revealed changes in the tricarboxylic acid cycle, accumulation of ketone body, and altered levels of key metabolites, such as 3-hydroxybutyric acid and serine, suggesting intricate links between energy metabolism and redox reactions. Plasma metabolomic analysis revealed alterations in mitochondrial metabolism, nicotinamide metabolism, and tryptophan degradation. The gut microbiome showed shifts, with higher PQ doses influencing microbial populations (e.g., *Escherichia coli* and *Akkermansia muciniphila*) and metagenomic functions (pyruvate metabolism, fermentation, nucleotide and amino acid biosynthesis). Overall, this study provides comprehensive insights into the complex interplay between PQ exposure, metabolic responses, and gut microbiome dynamics. These findings enhance our understanding of the mechanisms behind oxidative stress-induced metabolic alterations and underscore the connections between xenobiotic exposure, gut microbiota, and host metabolism.

## 1. Introduction

Paraquat (PQ) is a superoxide-generating chemical that has historically been used as an herbicide. However, since its commercialization in 1961, numerous cases of fatal PQ poisonings have been reported because of accidental or deliberate ingestion of concentrated PQ formulations [1]. PQ poisoning is one of the preferred methods of suicide in developing countries, marked by a significant global mortality rate of 20 deaths per million people [2]. While some regions, such as Europe, have implemented bans resulting in reduced suicide rates, some countries persist in using this herbicide [3]. Even sublethal doses or skin exposure can lead to serious lung and kidney damage [1]. During application, inhalation may result in pulmonary edema and impaired lung function [4], while skin contact can cause dermatitis [5]. Notably, paraquat use has been linked to chronic health issues, with recent studies confirming its association with Parkinson’s disease [6]. Clinically managing PQ poisoning presents a complex challenge due to high morbidity, mortality, and the lack of effective treatments in humans [1]. Much remains to be understood about the toxicology of PQ at both preclinical and clinical levels.

In addition to its use as an herbicide and as an intentional suicide poison, PQ is often used experimentally as an environmental inducer of oxidative stress [7]. At the toxicodynamic level, the main molecular mechanism of PQ toxicity is based on redox cycling and the generation of intracellular oxidative stress [8]. PQ undergoes reduction, mainly by NADPH-cytochrome P-450 reductase, NADPH-cytochrome c reductase and mitochondrial complex I, resulting in the formation of a monocation free radical of PQ (PQ^•+^). Subsequently, PQ^•+^ is rapidly re-oxidized in the presence of oxygen to form O_2_^•−^, which is physiologically generated during cellular respiration and arachidonic acid metabolism by the activity of LOX and COX, respectively [9]. This initiates the well-documented cascade leading to the generation of other ROS, such as H_2_O_2_ and HO^•^, which disrupt cellular homeostasis. This increased ROS production induces non-selective oxidation of essential biomolecules, including lipids, proteins, and nucleic acids, ultimately leading to cellular damage and cell death [7].

In this context, metabolic profiling emerges as a valuable tool for investigating toxicity, providing a unique mechanistic insight into toxicological responses. In recent years, metabolomics has found widespread application in the discovery of biomarkers and metabolic fingerprints, playing a crucial role in drug discovery and clinical toxicology [10]. This approach has been instrumental in exploring systematic metabolic responses to toxins and unravelling the mechanisms involved such as the case of PQ [11]. However, there are few studies targeting gut microbiome effects on PQ poisoning and none of them are in rats [12,13]. Moreover, the integration of metagenomics into this framework further enhances our understanding of the complex interactions within microbial communities and their influence on host metabolism [13], opening new avenues for comprehensive toxicity studies.

Hence, we aimed to explore the metabolic and gut microbiome responses in male Wistar rats following PQ administration, with a specific focus on the implications of oxidative stress. To achieve this, rats were treated with a single intraperitoneal administration of two different doses of PQ 15 (PQ-15) and 30 mg/kg (PQ-30), known to induce oxidative stress damage [14]. This study offers a holistic perspective on the intricate relationship between PQ, oxidative stress, and their impact on both the host metabolism and gut microbiome in male Wistar rats. These findings contribute to the knowledge of the molecular consequences of PQ intoxication at the metabolome and microbiome level, which could help to discover novel strategies for PQ-intoxication treatment.

## 2. Materials and Methods

### 2.1. PQ Administration in Male Wistar Rats

Thirty 8-week-old male Wistar rats (Harlan Laboratories, Barcelona, Spain) were housed individually under a fully controlled condition including temperature (22 ± 2 °C), humidity (55 ± 5%) and light (12 h light–dark cycle and lights on at 7:30 a.m.). All the procedures were approved by the Animal Ethics Committee of the University Rovira i Virgili (Tarragona, Spain) and the Generalitat de Catalunya (protocol code 10061). The study followed the ‘Principles of Laboratory Animal Care’, complied with the ARRIVE guidelines and was carried out in accordance with the EU Directive 2010/63/EU for animal experiments.

**Figure 1:**
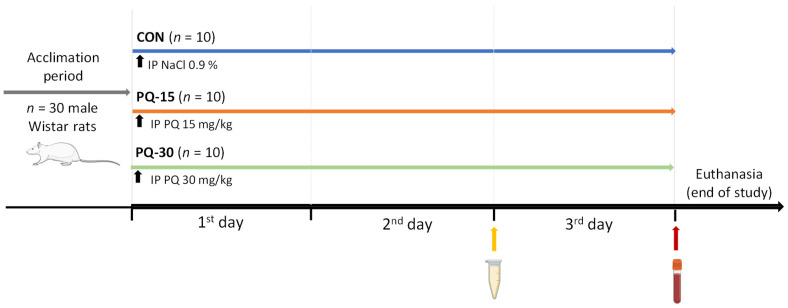
Schematic representation of the PQ-induced oxidative stress model. The experimental model consisted of three groups that received a single intraperitoneal injection of 15 and 30 mg/kg of PQ and vehicle/saline solution (NaCl 0.9%) indicated with a black arrow. Before the end of study, urine and blood were collected as well as tissue samples. Abbreviations: IP, intraperitoneal injection; CON, control group; PQ-15, paraquat 15 mg/kq; PQ-30, paraquat 30 mg/kg.

Following an acclimation period, rodents were randomly assigned to three groups (*n* = 10 animals per group). The experiment was conducted during the light-phase (8:30–10:00 am) and was carried out for 3 days (Figure 1). On the initial day, each animal received a single intraperitoneal injection specific to its assigned group. The experimental groups were administered PQ (1,1′-dimethyl-4,4′-bipyridinium dichloride hydrate, Sigma-Aldrich, Madrid, Spain) doses of 15 mg/kg (PQ-15) and 30 mg/kg (PQ-30), adjusted according to body weight. The control group (CON) received the vehicle/saline solution (0.9% NaCl), also adjusted based on individual body weight.

### 2.2. Sample Collection

On the second day after the PQ administration, urine was collected using the hydrophobic sand method [15]. For each rat, 300 g of hydrophobic sand (LabSand, Coastline Global, Palo Alto, CA, USA) was spread on the bottom of a plastic mouse cage. Urine was collected every half hour for 6 h. For preservation urine was mixed with sodium azide (Sigma, St. Louis, MO, USA) at the end of the session. 

On the third day after the PQ administration, rats were euthanized by guillotine under anesthesia (pentobarbital sodium, 50 mg/kg per body weight) after 7 h of fasting. Blood was collected and centrifuged at 3000× *g* at 4 °C for 15 min to recover plasma. Tissues were rapidly removed, weighed and snap-frozen in liquid nitrogen (i.e., retroperitoneal white adipose tissue (RWAT), mesenteric white adipose tissue (MWAT), muscle, liver, and cecum). Samples were stored at −80 °C until further analysis.

### 2.3. General Measurements for the Characterization of the Experimental Approach

#### 2.3.1. Determinations in Plasma

Enzymatic colorimetric kits were used for the general determination of plasma total cholesterol (TC), triglycerides (TG), glucose (QCA, Barcelona, Spain) and non-esterified free fatty acids (NEFAs, WAKO, Neuss, Germany). 

To evaluate oxidative stress, we measured the markers of lipid oxidative damage by measuring malondialdehyde (MDA, TBARS assay kit, Cayman Chemical Company, Ann Arbor, MI, USA) and 8-isoprostane (8-isoprostane ELISA kit, Cayman Chemical Company, Ann Arbor, MI, USA). To know the antioxidant capacity of the subjects, we quantified the activity of the main antioxidant enzyme, superoxide dismutase (SOD, SOD Colorimetric Activity Kit, Thermo Fisher Scientific, Waltham, MA, USA). The overall inflammatory response was measured with the level of the monocyte chemoattractant protein-1 (MCP-1, MCP-1 Rat Instant ELISA™ Kit, Thermo Fisher Scientific, Waltham, MA, USA) one of the main pro-inflammatory cytokines. The manufacturer’s protocol was followed for all the determinations.

#### 2.3.2. Measurement of ROS in Liver Homogenates

ROS levels were quantified in liver homogenate using dihydrodichlorofluorescein diacetate prove (DCF-DA, Sigma-Aldrich, Madrid, Spain). Approximately 50 mg of liver was homogenized with Tyssuelyser LT (Qiagen, Hilden, Germany) for 1 min at 50 oscillations/s in 500 µL of RIPA lysis buffer (0.5 M Tris-HCl, pH 7.4, 1.5 M NaCl, 2.5% deoxycholic acid, 10% NP-40, 10 mM EDTA). The liver homogenates were centrifuged at 1600× *g* for 10 min and the supernatant obtained was known as total liver homogenated and was stored at −80 °C for the assessment of protein levels and ROS. The liver homogenates were quantified by the standardized BCA method (Bio-RadProtein Assay; BioRad, Hercules, CA, USA). Briefly, 90 µL of liver homogenate (1:10) was dispensed into a 96 well black plate to which 10 mL of DCFDA was added to a final concentration of 10 µM and incubated for 30 min at room temperature to allow the cleavage of DCFDA by esterases and further conversion into the fluorescent product dichloro- fluorescein. The fluorescence was measured using a multimode plate reader (FLUOstar Omega, BMG LABTECH, Ortenberg, Germany) with excitation at 480 nm and emission at 530 nm. Results were normalized using total protein concentration (BCA protein assay, Thermo Fisher Scientific, Madrid, Spain) in each sample. 

#### 2.3.3. RNA Extraction and qPCR

Total RNA was obtained from liver samples using TriPure reagent (Roche Diagnostic, Barcelona, Spain) and RNeasy Mini Kit (QIAgen, Madrid, Spain) as described in supplier’s protocol. RNA concentration and purity were measured by the determination of the absorbance at 260 and 280 nm with a nanophotometer (Implen, Munich, Germany). RNA was converted to cDNA using a High-Capacity cDNA Reverse Transcription Kit (Applied Biosystems, Wilmington, DE, USA) with a RNase Inhibitor (Applied Biosystems, Wilmington, DE, USA) as described in manufacturer’s protocol.

The gene expression related to oxidative stress (i.e., *Cu/Zn SOD, Mn SOD, CAT, GPx1*) were evaluated by quantitative polymerase chain reaction (qPCR). For this purpose, cDNAs samples were diluted 1:10 before being incubated with commercial LightCycler^®^ 480 SYBR Green I Master on a LightCycler^®^ 480 II (Roche Diagnostics, Manheim, Germany). Table 1 shows a list of primers used and the selected housekeeping gene was *PPIA*. The primers were synthetized by Biomers.net (ULM, Germany). Thermal cycling comprised an initial step at 95 °C for 5 min and a cycling step with the following conditions: 45 cycles of denaturation at 90 °C for 10 s, annealing at 60 °C for 10 s and extension at 72 °C for 10 s. Fluorescence data were acquired at 72 °C of cycling step.

#### 2.3.4. Protein Extraction and Western Blot Analysis

Liver samples were homogenated in RIPA buffer (50 mM Tris-HCL, 150 mM NaCl; pH 7.4, 1% Tween 20, 0.25% Na-deoxycholate) containing phenylmethylsulfonyl fluoride (PMSF, Sigma-Aldrich, Madrid, Spain), phosphatase cocktails 2 and 3 (Sigma-Aldrich, Madrid, Spain) and protease inhibitor cocktail (PIC, Sigma-Aldrich, Madrid, Spain) with TissueLyser LT (QIAgen, Madrid, Spain) for 50 s. After shaking samples for 30 min at 4 °C, the lysates were centrifuged at 16,300× *g* for 15 min at 4 °C. Finally, protein concentration was measured by bicinchoninic acid (BCA) protein assay kit (Thermo Fisher Scientific, Madrid, Spain). 

Briefly, 50 μg of protein per sample were electrophoretically separated on 10% SDS-polyacrylamide gels (TGX FastCast Acrylamide Kit, Bio-Rad, Madrid, Spain) and transferred to PVDF membranes (Trans-Blot Turbo System, Bio-Rad, Barcelona, Spain). Protein transfer efficiency was evaluated by Pounceau-S stain. Then, membranes were blocked with 5% non-fat milk in TBS-Tween (0.2%) for one hour at room temperature. After blocking, membranes were blotted overnight at 4 °C with rabbit antibodies (dilution 1:1000): Monoclonal antibody for Glutathione Reductase (Abcam, Cambridge, UK) and Monoclonal antibody for β-Actine (Abcam, Cambridge, UK) were used. After 3 washings with TBS-Tween, membranes were hybridized with anti-rabbit secondary antibody (Cell signaling, Amersham, Cytiva, Barcelona, Spain) (dilution 1:10,000) conjugated with horseradish peroxidase for 1 h at room temperature. After 3 more washings, immunoreactive proteins were visualized using a chemiluminescence substrate kit (Amersham ECL Select, Cytiva, Barcelona, Spain) following the supplier’s protocol. Digital images were obtained with a G:BOX Chemi XL1.4 (Syngene, Cambridge, UK) and densitometry analysis were evaluated using ImageJ Software 1.54g (NIH, Bethesda, MD, USA).

### 2.4. Plasma Metabolomics (GC-qTOF and UHPLC-qTOF)

Plasma metabolites were determined by gas Chromatography coupled with Quadrupole Time-of-Flight (GC-qTOF). To extract the metabolites, a protein precipitation method was used in which a solution of methanol:water (8:2) containing internal standards (succinic acid-d_4_, myristic acid-d_27_, glycerol-^13^C_3_ and D-glucose-^13^C_6_) was added to plasma samples. The samples were then mixed, incubated at 4 °C for 10 min, centrifuged at 21.420× *g* and the resulting supernatant was evaporated before compound derivatization (metoximation and silylation). GC-qTOF (Agilent, USA) was used to analyze the derivatized compounds, using the Fiehn Method for chromatographic separation with a J&W Scientific HP5-MS column (30 m × 0.25 mm i.d.). Electronic impact (EI) at 70 eV was used for ionization, operating in full scan mode. Metabolite identification was based on comparison of EI mass spectrum and retention time with the Fiehn metabolomics library (Agilent, Santa Clara, CA, USA). After putative identification, metabolites were semi-quantified using internal standard response ratio.

Ultra High-Performance Liquid Chromatography coupled with Quadrupole Time-of-Flight (UHPLC-qTOF) was used for the analysis of plasma lipids. Hydrophobic lipids were extracted by the Folch method. A mixture of chloroform: methanol (2:1) containing an internal standard mixture (Lipidomic SPLASH^®^, Avanti Polar Lipids, Inc., Alabaster, AL, USA) was added to the plasma. Followed by incubation at −20 °C for 30 min. Water with NaCl (0.8%) was then added and the resulting mixture was centrifuged at 21.420× *g*. The lower phase was collected, evaporated to dryness and reconstituted with methanol: methyl-tert-butyl ether (9:1) for analysis by UHPLC-qTOF (Agilent, USA) in positive electrospray ionization mode. The chromatography was performed using a quaternary mobile phase of water (A), methanol (B), 2-propanol (C) and water with 200 mM ammonium formate and 2% formic acid (D). The gradient was as follows: 0–0.5 min, 40% A, 10% B, 45% C; 0.5–1.5 min, 37.8% A, 9.5% B, 47.7% C; 1.5–1.6 min, 28.7% A, 7.5% B, 58.8% C; 1.6–5 min, 26.8% A, 7% B, 61.2% C; 5–5.1 min, 13.6% A, 4% B, 77.4% C; 5.1–7.5 min, 11.4% A, 3.5% B, 80.1% C; 7.5–9 min, 11.4% A, 3.5% B, 80.1% C; 9–9.5 min, 95% C; 9.5–11.5 min, 95% C; 11.5–11.6 min, 40% A, 10% B, 45%C. The separation process was performed on a C18 column (Kinetex EVO C18 Column, 2.6 µm, 2.1 mm × 100 mm) at 60 °C. This temperature facilitates the sequential elution of highly hydrophobic lipids, including triglycerides (TGs), diacylglycerols (DGs), phosphatidylcholines (PCs), cholesterol esters (ChoEs), lysophospholipids (LPCs) and sphingomyelins (SMs). Lipid species were identified by comparison of their accurate mass and, where available, tandem mass spectra with Agilent’s Metlin-PCDL. In addition, putative identification was ensured by considering the chromatographic behavior of pure standards from each lipid family and the relevant literature information. These standards included 1-stearoyl-rac-glycerol (Sigma-Aldrich, Madrid, Spain), 1-steraroyl-2-hydroxy-sn-glycero-3-phosphocholine (Avanti Polar Lipids, Inc., Alabaster, AL, USA), 1,2-dipalmitoyl-sn-glycero-3-phosphocholine (Avanti Polar Lipids, Inc., Alabaster, AL, USA), sphingomyelin (Avanti Polar Lipids, Inc., Alabaster, AL, USA), 1,2-dipalmitoyl-sn-glycero-3-phosphoethanolamine (Avanti Polar Lipids, Inc., Alabaster, AL, USA), 1,2-dioctadecanoyl-sn-glycerol (Avanti Polar Lipids, Inc., Alabaster, AL, USA), 1-palmitoyl-2-oleoyl-3-linoleoyl-rac-glycerol (Sigma-Aldrich, Madrid, Spain) and cholesteryl palmitate (Sigma-Aldrich, Madrid, Spain). Following putative lipid identification, semi-quantification of lipids was performed in terms of internal standard response ratio, using one internal standard for each lipid family.

A pooled matrix of samples, acting as a technical replicate of the entire dataset, was generated by extracting a small volume from each experimental sample. As the study spanned several days, a data normalization step was performed to correct for variation due to instrumental differences. 

### 2.5. Urine Metabolome (^1^H-NMR)

Proton Nuclear Magnetic Resonance (^1^H-NMR) was used to evaluate urinary metabolites. The urine sample was mixed (1:1) with phosphate buffered saline containing with 3-(trimethylsilyl)propionic-2,2,3,3-d_4_ acid sodium salt (TSP) (Sigma Aldrich, Madrid, Spain) and placed in a 5 mm NMR tube for direct analysis by ^1^H-NMR. The ^1^H-NMR spectra were recorded at 300 K on an Avance III 600 spectrometer (Bruker^®^, Bremen, Germany) operating at a proton frequency of 600.20 MHz using a 5 mm Broad Band Observe Probe (PBBO). Diluted urine samples were measured and spectral information was acquired in procno 11 by One-dimensional ^1^H pulse experiments using nuclear Overhauser effect spectroscopy (NOESY). The NOESY presaturation sequence (RD–90°-t1-90°-tm-90° ACQ) was used to suppress the residual water peak with a mixing time of 100 ms. Solvent presaturation with an irradiation power of 150 μW was applied during the recycling delay (RD = 5 s) and mixing time using noesypr1d pulse program (Bruker^®^, Bremen, Germany) to eliminate residual water. The 90° pulse length was calibrated individually for each sample at approximately 11 microsec. The spectral width was 9.6 kHz (16 ppm) with 128 transients collected into 64 k data points for each ^1^H spectrum. Prior to Fourier transformation, an exponential line broadening of 0.3 Hz was applied. The spectra in the frequency domain were subjected to manual phasing and baseline correction using TopSpin software (v.3.2, Bruker, Bremen, Germany). Normalization was performed using probabilistic methods to mitigate differences between samples due to varying urine concentrations and ERETIC. The resulting ^1^H-NMR data were compared with pure compound references from the AMIX metabolic profiling spectra database (Bruker^®^, Bremen, Germany), HMDB and Chenomx databases for metabolite identification. In addition, metabolites were assigned by ^1^H-^1^H homonuclear (COSY and TOCSY) and ^1^H-^13^C heteronuclear (HSQC) 2D NMR experiments and by correlation with in-house pure compounds. After preprocessing, specific ^1^H-NMR regions identified in the spectra were integrated using in-house MATLAB scripts. 

### 2.6. Shotgun Metagenomics Sequencing

DNA was extracted from cecal content using the PowerSoil DNA extraction kit (MO BIO Laboratories, Carlsbad, CA, USA) following the manufacturer’s protocol. Between 400 and 500 ng of total DNA was used for library preparation for Illumina sequencing employing Illumina DNA Prep kit (Illumina, San Diego, CA, USA). All libraries were assessed using a TapeStation High Sensitivity DNA kit (Agilent Technologies, Santa Clara, CA, USA) and were quantified by Qubit (Invitrogen, Waltham, MA, USA). 

Validated libraries were pooled in equimolar quantities and sequenced as a paired-end 150-cycle run on an Illumina NextSeq2000 (Illumina, San Diego, CA, USA). A total of 1548 million reads were generated, and raw reads were filtered for QV > 30 using an in-house phyton script. Filtered reads were aligned to unique clade-specific marker genes using MetaPhlAn 3 [16] to assess the taxonomic profile. Alignment was performed indicating the closest name of species to the sequence (the best hit). The relative proportions calculated from MetaPhlAn were used to calculate relative abundances, alpha diversity measure (chao1 index) and beta diversity measures (Aitchison distance). 

### 2.7. Statistical Analysis

#### 2.7.1. General Statistical Analysis

The results were expressed as the mean ± SEM with statistical comparisons carried out using one-way ANOVA, followed by the study of the normality and Tukey’s multiple comparison test. In all the statistical comparisons, a two-tailed *p*-value < 0.05 was considered. Across the different statistical analysis, the magnitude of difference between populations is presented as fold change (FC). The statistical analysis was performed using different software: (1) the R statistical software v4.0.2 (R Core Team 2021) and different libraries, included in Bioconductor v3.11 (Bioconductor project) as rolps and mixOmics; (2) SPSS v25.0 (SPSS Inc., Chicago, IL, USA); (3) GraphPad Prism v8.0.0 (GraphPad Software, San Diego, CA, USA).

#### 2.7.2. Metabolomic Data Analysis

Individual comparisons between metabolites were determined by the Kruskal–Wallis H-test, a non-parametric version of ANOVA. The *p*-value adjustment for multiple comparisons was carried out according to the Benjamin–Hochberg (BH) correction method with a false discovery rate (FDR) of 5%, and a post hoc Dunn. In parallel, a predictive analysis was run to evaluate the prediction power of the oxidative stress model. On the one hand, principal component analysis (PCA), an unsupervised multivariate data projection method, was performed to explore the native variance of the samples. On the other hand, partial least squares discriminant analysis (PLS-DA) was performed to determine the prediction power that supervised multivariate data projection method explores, relationships between the observable variables (X) and the predicted variables or target (Y) by regression extensions. The predictive performance of the test set was estimated by the Q2Y parameter calculated through cross-validation. The values of Q2 < 0 suggests a model with no predictive ability, 0 < Q2 < 0.5 suggests some predictive character and Q2 > 0.5 indicates good predictive ability [17]. The feature importance was calculated through the variable importance in projection (VIP), which reflects both the loading weights for each component and the variability of the response explained by the component.

#### 2.7.3. Metagenomic Data Analysis

Centered log-ratio (CLR) normalization was performed before any statistical test. The beta diversity was calculated from the Aitchison distance and PERMANOVA test was performed with 100 permutations to assess the differences between groups. The alpha diversity was calculated by Chao1 index. Taxonomic abundances, which are presented by relative abundance (%), were compared between experimental groups using the Holm-Šídák (HS) post hoc adjustment on Kruskal–Wallis test. The relative abundance was filtered to only include variables that were present above 0.01% in at least 3 samples [18]. 

#### 2.7.4. Multi-Omics Data Association with Isoprostanes (Gold Standard Biomarker)

To find significant associations between isoprostanes and specific features of the multi-omics data generated, the multi-omics data were input to the MaAslin2 comprehensive R package (Multivariate Association with Linear Models 2, v.1.8.0—Bioconductor) alongside isoprostanes levels. Results with a q-value of <0.25 were considered significant [19]. 

#### 2.7.5. Integration Data Analysis

Multiblock sPLS-DA, which is also known as Data Integration Analysis for Biomarker discovery using Latent cOmponents (DIABLO), is a holistic approach with the potential to find new biological insights not revealed by any single-data omics analysis, as some pathways are common to all data types, while other pathways may be specific to data. DIABLO is built on the Generalized Canonical Correlation Analysis (GCCA) [20] in the mixOmics R package [21] (v.6.18.1, mixOmics project) and in our case it was used to integrate the plasma and urine metabolome and the microbiome. 

To summarize, the first step is the parameter choice of the design matrix, the number of components and the number of variables to select: (1) To improve accuracy, a design matrix based on pairwise correlations was performed; (2) the perf function was used to estimate the performance of the model and the balanced error rate (BER) and overall error rates per component were displayed to select the optimal number of components; (3) the number of variables were chosen using the tune.block.splsda function that was run with 10-fold cross validation and repeated 10 times. Thereafter, the final model was computed, and different sample and variable plots performed. The circosPlot function represents the correlations between variables of diverse types, represented on the side quadrants that are built based on a similarity matrix [22]. 

The final performance of the model was evaluated by the perf function using 10-fold cross-validation repeated 10 times. The receiver operating characteristic (ROC) curve analysis was conducted to determine the optimal metabolite combination patterns that could correctly dichotomize the stressed and healthy groups at acceptable sensitivity and specificity (defined as greater than 80% for both). The area under the ROC curve (AUC) value was used as a measure of the prognostic accuracy. 

#### 2.7.6. Pathway Analysis

The resulting significant differential features were analyzed through different data bases to identify related pathways and elucidate the global effect in the metabolome of the LPS-induced inflammation model. The main data base consulted was the Kyoto Encyclopedia of Genes and Genomes (KEGG). To visualize results and incorporate information regarding pathway analysis, a mapping tool (XMind 2020, v.XMind 2020, XMind Ltd., Virginia, ON, Canada) was used.

## 3. Results

### 3.1. Impact of PQ Administration on Physiological and Biochemical Responses

#### 3.1.1. Body Changes and General Parameters in Rats

The administration of PQ influenced the animals in terms of body weight and general biochemical changes, which are summarized in Table S1. The final body weight was significantly decreased in the PQ groups compared to the CON group. In particular, a substantial reduction in the final body weight was observed in PQ-30 compared to CON. In this context, PQ administration resulted in a significant reduction in food intake. Both the PQ-15 and PQ-30 groups have shown significant reductions to approximately a half of its weight in PQ-15 and almost to a fasting levels in PQ-30, compared to the CON group. Tissue weights indicated changes in adipose tissue and muscle, with a consistent decrease in liver and cecum weights. Other general parameters of interest, such as plasma TG, tended to decrease in both PQ groups compared to CON, whereas TC was significantly increased. Despite these changes, no significant differences in plasma glucose and NEFA levels were observed between the groups.

#### 3.1.2. Oxidative and Inflammatory Profiling in Liver, Plasma and Urine

**Figure 2:**
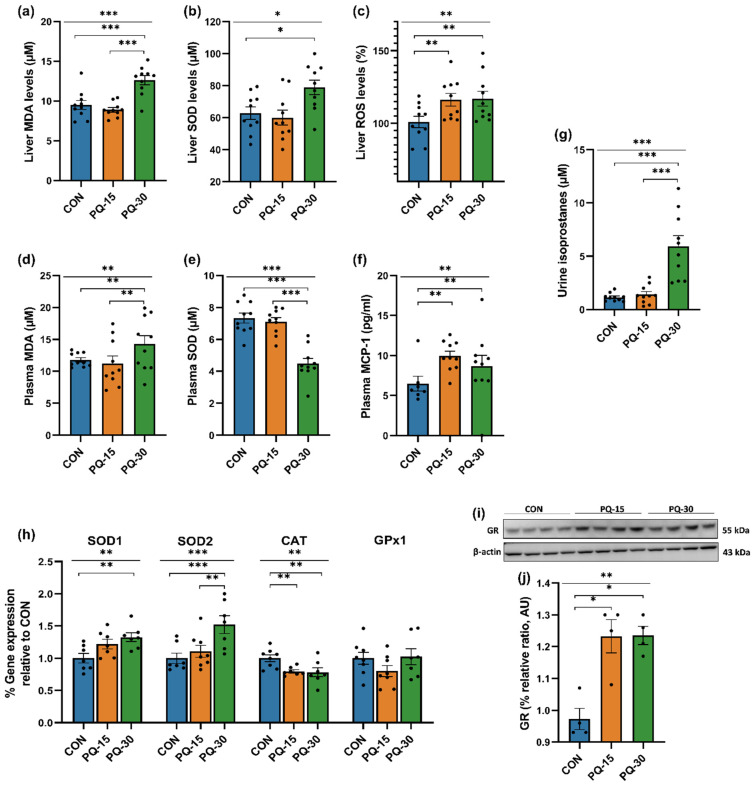
Advanced insights into PQ-induced oxidative stress and inflammatory responses. Liver concentrations of MDA, SOD and ROS levels (**a**–**c**), plasma concentrations of MDA, SOD and MCP-1 levels (**d**–**f**), urine concentrations of 8-isoprostanes (**g**), hepatic mRNA expression of SOD1, SOD2, CAT and GPx1 (**h**), Western blotting of GR from hepatic proteins including image (**i**) and densitometric analysis normalized to β-Actin (**j**). The results are presented as the mean ± SEM (*n* = 4–10 animals per group). The statistical comparisons among groups were conducted using one-way ANOVA and post hoc (Tukey) test. * Denotes *p* < 0.1 (tendency), ** *p* < 0.05 (significantly different) and *** *p* < 0.01 (high significantly different) compared with control. Abbreviations: CON, control; PQ-15, paraquat 15 mg/kg; PQ-30, paraquat 30 mg/kg; MDA, malondialdehyde; SOD, superoxide dismutase; ROS, reactive oxygen species; MCP-1, monocyte chemoattractant protein-1; CAT, catalase; GPx1, glutathione peroxidase 1; GR, glutathione reductase.

In the PQ-30 group, a significant elevation of MDA and SOD was observed in liver tissue, underscoring heightened oxidative stress (Figure 2a,b). Additionally, ROS was elevated in both doses confirming the induction of oxidative stress (Figure 2c). Plasma MDA increased in both doses while only SOD decreased in PQ-30 (Figure 2d,e). After the PQ exposure, the proinflammatory cytokine MCP-1 increased in contrast to the control group (Figure 2f). Furthermore, the urine 8-isoprostane level, being classified as the “gold standard” in estimating oxidative injury, was increased in the PQ-30 group (Figure 2g). Hepatic gene expression involved in inflammatory pathway was investigated (Figure 2h). On the one hand, the cytoplasmatic SOD (*SOD1* or *Cu/Zn SOD*), shows a significant increase at both doses of PQ, being statistically significant at the higher dose. Moreover, a similar result is presented by mitochondrial SOD (*SOD2* or *Mn SOD*), which shows statistically significant differences at the highest dosage. On the other hand, the expression of the *CAT* gene, decreased statistically in both doses regarding with control group (Figure 2c). Moreover, we assessed a Western blotting analysis of glutathione reductase (GR), determinant enzyme for the GSSG/GSH ratio (defense against ROS). The results obtained showed treatment with PQ significantly increased the total amount of GR enzyme, obtaining a similar response after both doses of treatment (Figure 2i,j and Figure S1). Collectively, these results indicated that PQ induced changes in oxidative stress and inflammation at different levels.

### 3.2. Exploring Plasma Metabolomic Alterations in Response to PQ Administration

Our study employed a comprehensive plasma metabolomics approach that is based on a multiplatform global analysis (GC-qTOF and UHPLC-qTOF), evaluating the relative abundance of 128 metabolites with highest impact on metabolome (Table S2). We obtained 109 metabolites with a different mean between groups by Kruskal–Wallis H-test. Following false discovery rate correction, 107 metabolites had at least two groups with different means. After the post hoc test to check which of the 3 relations was causing the difference in the mean, 74 metabolites were different between CON and PQ-15; 70 metabolites were different between CON and PQ-30; and 58 metabolites were different between PQ-15 and PQ-30 (Figure S2). 

**Figure 3:**
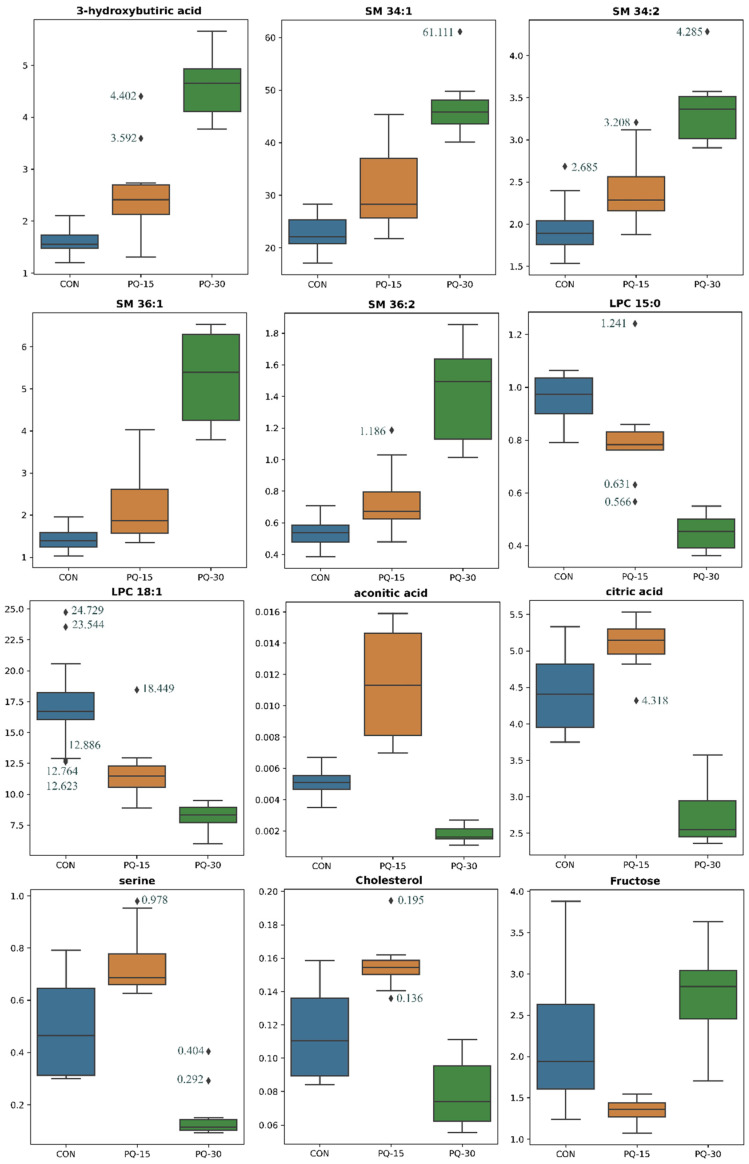
Boxplots of the 12 plasma metabolites significantly different between CON, PQ-15 and PQ-30. Box denotes 25th and 75th percentiles; line within box denotes 50th percentile (median); whisker denotes standard deviation. Groups (*n* = 10 animals per group): CON, blue; PQ-15, orange; PQ30, green. Abbreviations: SM, sphingomyelin; LPC, lysophospholipid.

As shown in Figure 3, 12 metabolites—aconitic acid, fructose, cholesterol, citric acid, 3-hydroxybutiric acid, serine, LPCs (LPC 18:1 and LPC 15:0) and SMs (SM 34:1, SM 34:2, SM 36:2 and SM 36:1)—significantly varied across all groups. When visualized using box plots (Figure 3), the distribution patterns of these metabolites showed intriguing deviations from the expected dose–response trend. In particular, aconitic acid, citric acid, serine, cholesterol and fructose deviated from this pattern, warranting further extensive comparisons to unravel the metabolic implications. 

When comparing the control group with PQ-15, prominent alterations encompassed ten high-impact lipid metabolites, primarily including diverse TGs such as TG 48:3, TG 48:1, TG 48:2, TG 46:1, TG 46:0, TG 52:6 and TG 50:4, as well as specific lipids as DG 34:3, PC 38:3 and SM 35:1. In the case of control versus PQ-30, the 10 metabolites altered with high impact were two groups of lipids, which are LPCs (LPC 18:1, LPC 18:2 and LPC 15:0) and SMs (SM 34:1, SM 34:2, SM 36:1, SM 36:2 and SM 38:1), and a ketone body (i.e., 3-hydroxybutiric acid). Comparing PQ-15 and PQ-30 groups, variations emerged in intermediate metabolites of the TCA cycle (i.e., aconitic acid, citric acid and fumaric acid), cholesterol, serine, fructose, glycolytic acid and ChoE (22:5), among others.

PCA explains the variance in the plasma metabolome, showing that the data tend to be segregated in the three different groups (Figure S3a). Additionally, PLS-DA was performed to assess the discriminative power of the different groups (Figure S3b). The proportion of variance explained by the model (R2X) was 47% in the plasma data. The percentage of Y variability explained by the model (R2Y) was 65,5% and, the estimation of the predictive performance of the models (Q2) was 57,8%. A model is considered to have good predictability when the Q2 is greater than 50%, thus the predictive power of the model in plasma was good. The metabolites with the highest VIP values, which reflects both the loading weights for each component and the variability of the response explained by this component, coincide with the 12 differential metabolites between groups and presented values near to 2 (Table S2).

### 3.3. Exploring Urine Metabolomic Alterations in Response to PQ Administration

Our urine metabolomics approach is based on H1-NMR that method determined the relative abundance (AU) of 21 metabolites (Table S3). We identified 15 metabolites having at least two groups with different means by Kruskal–Wallis H-test and corrected by Benjamin/Hochberg method. After the post hoc test to check from which of the 3 relations was causing the difference in the mean, 4 metabolites were different between CON and PQ-15; 13 metabolites were different between CON and PQ-30; and 10 metabolites were different between PQ-15 and PQ-30 (Figure S4).

**Figure 4:**
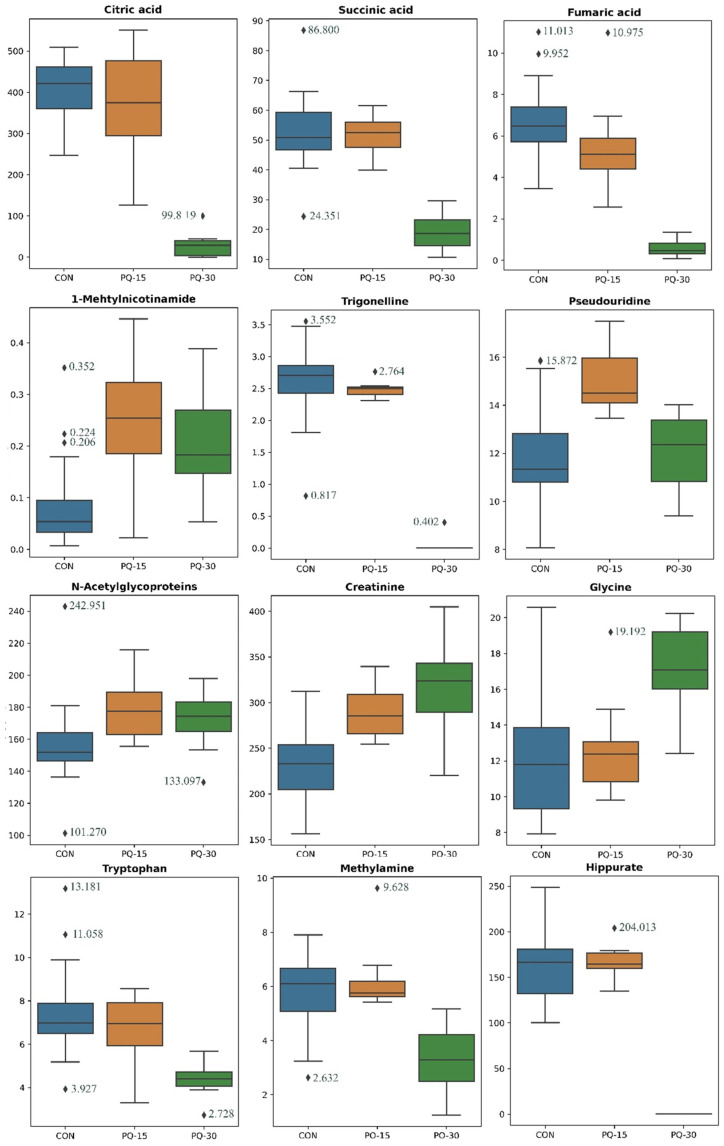
Boxplots of the 12 urine metabolites significantly different in pair-wise comparisons between CON, PQ-15 and PQ-30. Box denotes 25th and 75th percentiles; line within box denotes 50th percentile (median); whisker denotes standard deviation. Groups (*n* = 10 animals per group): CON, blue; PQ-15, orange; PQ-30, green.

The common metabolites between the pair-wise comparisons are shown in Figure 4. The differences between CON and PQ-15 groups were attributed to the levels of creatinine, pseudouridine, 1-methylmicotinamide and N-acetylglycoproteins. Differences between CON and PQ-30 groups mirrored those previously detected in CON vs. PQ-15 (creatinine and N-acetylglycoproteins), TCA intermediates (fumaric acid, citric acid, and succinic acid), nicotinamide intermediates (1-methylnicotinamide and trigonelline), amino acids (tryptophan, glycine, alanine and hippurate) and lactate. Finally, the differences associated to the different doses (PQ-15 and 30 mg/kg) were similar to the differences of CON against PQ-30 group including the TCA intermediates, nicotinamide intermediates, some previously described amino acids (tryptophan, glycine and hippurate) including a derivate of glycine (N,N-dimethylglycine) and pseudouridine. 

PCA shows that the variance if the data was able to discriminate the PQ-30 group in front the others (Figure S5a). Furthermore, PLS-DA was performed to assess the discriminative power of the different groups (Figure S5b). The proportion of variance in the urine data explained by the model (R2X) is 52.6%. The percentage of Y variability explained by the model (R2Y) is 66.1%, and the estimation of the predictive performance of the models (Q2) is 58.1%. However, we observed the same tendency as the PCA showing that PQ-30 segregates, and PQ-15 and CON are close. The main metabolites with the highest VIP values, which reflects both the loading weights for each component and the variability of the response are explained by this component (Table S3).

### 3.4. Impact of PQ-Administration on Gut Microbiome

#### 3.4.1. Taxonomic Impact of PQ-Administration

The metagenomic analysis characterized the effect of PQ-induced oxidative stress on the microbiome of the cecum section to evaluate the highest variability and diversity of the gut tract. The taxonomic assignment makes it possible to detect the presence of bacteria and viruses. In the control group, most of the generated readings (76%) were attributed to bacteria, with the remainder attributed to viruses. This trend persisted in the PQ-15 group, wherein 73% of readings were assigned to bacteria. in contrast, the PQ-30 group exhibited a shift, with 95% of readings associated to bacteria. The differences between the PQ-30 group and the others proved statistically significant (*p*-value < 0.02). 

**Figure 5:**
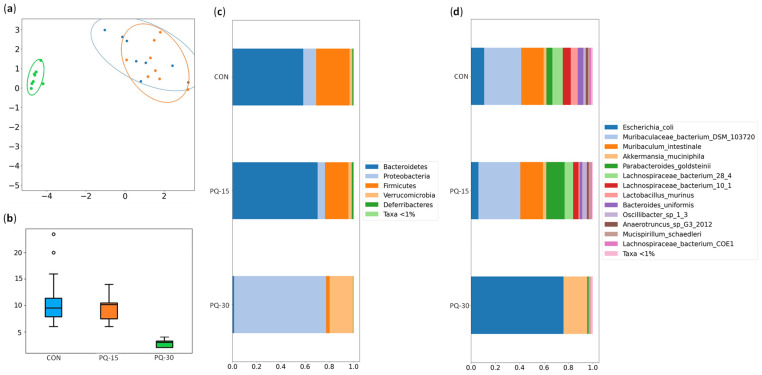
Summary of the bacteria statistical analysis in the PQ-induced oxidative stress model. (**a**) Beta diversity: PCA plot calculated by Aitchison distance. (**b**) Alpha diversity (AU): Chao1 index. (**c**) Relative distribution of bacterial phylum. (**d**) Relative distribution of bacterial species. Groups (*n* = 8 animals per group): CON, blue; PQ-15, orange; PQ-30, green.

The representation of the bacterial communities through the PCA (beta diversity) showed distinct differences in the PQ-30 group compared to the other groups (Figure 5a). Additionally, the PERMANOVA supports these observations, revealing significant statistical differences (F = 13.51, *p*-value < 0.01). The contrasting bacterial composition/beta diversity within this group were evident. The comparison of alpha diversity values (index that measures the richness of the sample) showed a clear decrease in chao 1 index in the PQ-30 group (Figure 5b), as it has been already observed in the beta diversity (Figure 5a).

In terms of bacterial diversity, the communities of the CON and PQ-15 groups are formed by the phylum *Bacteroidetes* (CON: 58% and PQ-15: 70%) and *Firmicutes* (CON: 27% and PQ-15: 19%) while the PQ-30 group is dominated by *Proteobacteria* (76%) and *Verrucomicrobes* (19%) (Figure 5c). The relative proportions of all phyla reported by the PQ-30 group are statistically different from those reported in the other groups (Table S4). Focusing on species, 27 species were found with a relative abundance above 0.01% at least in one group (Figure 5d, Table S5). In the CON and PQ-15 groups the species *Muribaculum intestinale* and *Murobaculaceae bacterium* predominate above the others. In contrast, *Escherichia coli* and *Akkermansia muciniphila* predominate in the PQ-30 group, possibly due to a decline in all of the other species. Overall, the PQ-30 group is statistically different from the other two groups and the main differences are outlined in Table 2. 

The representation of the virus communities in the PCA (beta diversity) shows that the communities of the different groups were similar (Figure S6a). In this line, the PERMANOVA test (F = 0.80, *p*-value > 0.05) indicates that there were no differences in bacteria composition/beta diversity. The comparison of Chao1 (index that measures the richness of the sample) indicated that the different groups presented similar alpha diversity (Figure S6b) following the same tendency as beta-diversity (Figure S6a). In terms of virus diversity, the communities of virus are mainly formed by the order of *Herpesvirales*, *Ortevirales* and *Caudovirales* (Figure S6c) without reporting differences between groups. Focusing on species, 18 species were found with a relative abundance above 0.01% in at least one group (Table S6). We observed that some residual viruses tend to differ between group; however, these differences were not considered due to no differences in beta diversity being found, and those residual differences could be associated to technical variability. In this case, *Cyprinid hespervirus 3* was the highest represented virus in all groups.

#### 3.4.2. Functional Impact of PQ-Administration

We identified 37 metagenomic functions with a representation higher than 1% at least in one of the experimental groups, as detailed in Figure S7 and Table S7. These findings highlight significant differences in the metabolic pathways among the CON, PQ-15, and PQ-30 groups. The alterations observed in metabolic functions appeared to exhibit dose-dependent effects, with greater changes in PQ-30 compared to PQ-15. 

The most relevant metagenomic functions were categorized based on their involvement in metabolic processes: 

Pyruvate metabolism and fermentation: The function PWY-7111, related to pyruvate fermentation to isobutanol, showed a significant decrease in PQ-30 compared to CON and PQ-15. This suggests that a high dose paraquat exposure may impact microbial fermentation pathways. 

Nucleotide Biosynthesis: Functions related to nucleotide biosynthesis (e.g., PWY-7219, PWY-6122, PWY-6277) displayed significant alterations in response to PQ exposure. These pathways may influence the availability of nucleotides for DNA and RNA synthesis. 

Amino Acid Biosynthesis: Several pathways involved in amino acid biosynthesis, including L-valine (VALSYN-PWY) and L-isoleucine (ILEUSYN-PWY) biosynthesis, were affected by paraquat exposure. These pathways contribute to the synthesis of essential amino acids and may impact host metabolism.

In summary, the metagenomic functional alterations in response to paraquat exposure indicate significant shifts in metabolic pathways related to energy metabolism, nucleotide biosynthesis, amino acid biosynthesis, and salvage pathways. These alterations may reflect adaptive responses of the gut microbiota to oxidative stress induced by paraquat exposure at different doses.

### 3.5. Correlation of Isoprostane Levels with Multi-Omics Data Reveals Oxidative Injury Induced by PQ Administration

**Figure 6:**
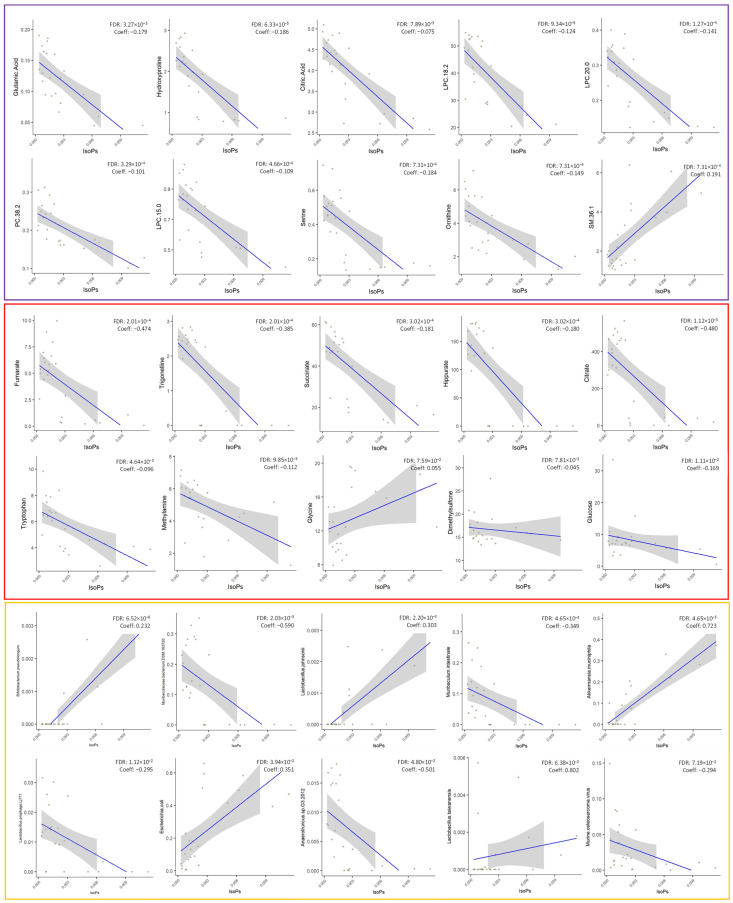
Top 10 plotting associations from most to least significant correlations between isoprostanes (isoPs) and multi-omics data. The multi-omics data were given as input to MaAslin2 comprehensive R package (Multivariate Association with Linear Models 2, v.1.8.0-Bioconductor) alongside isoprostanes levels. Results were considered significant if they had a *q*-value smaller than 0.25. Legend: Purple, plasma metabolites; red, urine metabolites; yellow, microorganisms.

We identified significant associations between isoprostane levels and various features within the multi-omics dataset. These associations are summarized in Table S8, which includes information about the feature type, specific feature, coefficient (Coef), standard error (stderr), *p*-value, and q-value. This gold standard biomarkers of oxidative injury were correlated with 67 plasma metabolites (24 positive and 43 negative correlated), 12 urine metabolites (3 positive and 9 negative correlated), and 16 microorganisms (5 positive and 11 negative correlated) (Table S8). The top 10 features in each dataset are represented in Figure 6.

In the plasma metabolome, metabolites such as glutamic acid, hydroxyproline, citric acid, LPC species, serine, and ornithine exhibited negative coefficients, indicating a negative correlation with isoprostane levels. In contrast, several metabolites, including valine, isoleucine, SMs species, and ChoE species, displayed positive coefficients, suggesting a positive correlation with isoprostanes.

In the urine metabolome, fumarate, trigonelline, succinate, hippurate, citrate, and tryptophan displayed negative coefficients, indicating an inverse relationship with isoprostane levels. In contrast, glycine, dimethylsulfone, 1-methylnicotinamide, and N,N-dimethylglycine exhibited positive coefficients, implying a positive correlation with isoprostanes.

Our analysis also revealed significant associations with various microorganisms. *Bifidobacterium pseudolongum*, *Lactobacillus johnsonii*, *Akkermansia muciniphila*, and *Escherichia coli* showed positive coefficients, suggesting a positive correlation with isoprostane levels. Conversely, *Muribaculaceae bacterium DSM 103720*, *Muribaculum intestinale*, *Anaerotruncus* sp. *G3.2012*, *Lachnospiraceae bacterium 10.1*, and *Bacteroides uniformis* displayed negative coefficients, indicating an inverse relationship with isoprostanes.

### 3.6. Multi-Omics Data Integration

Previously, regression analyses were performed with PLS to deeper understand the cross-correlation between different omics datasets. The pairwise correlations among datasets proved remarkable correlations: plasma and urine metabolome (r = 0.93); plasma metabolome and microbiome (r = 0.97); urine metabolome and microbiome (r = 0.91). These correlations are used to tune the final DIABLO model that is constructed with two components with a low error rate. Optimal variable selection based on these components revealed 6 and 9 plasma metabolites, 20 and 5 urine metabolites, and 5 and 30 microbes for the first and second component, respectively.

**Figure 7:**
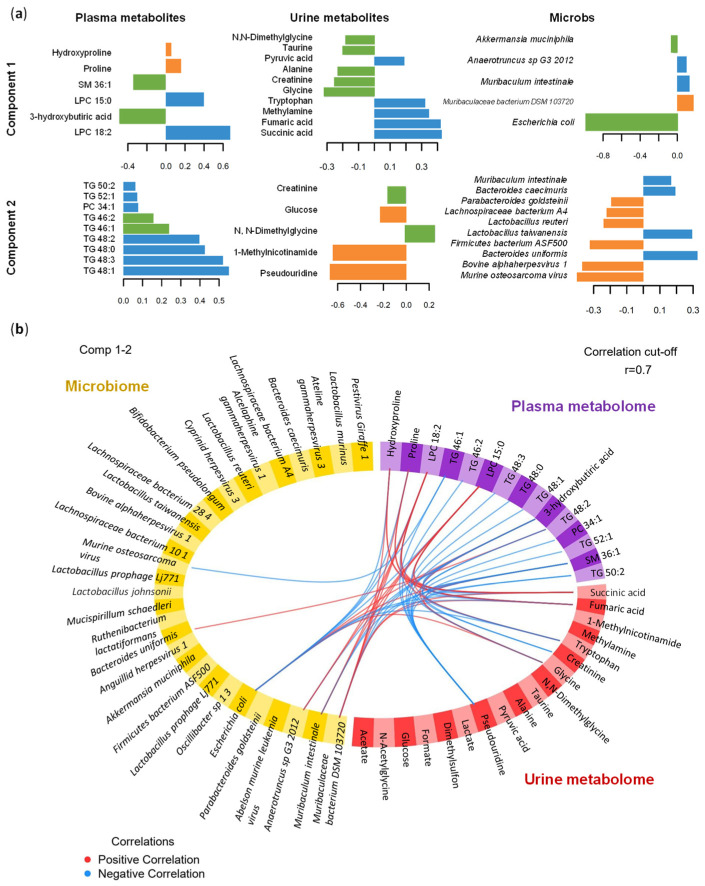
Multi-omics data integration of plasma metabolome, urine metabolome and microbiome using DIABLO in the oxidative stress model. (**a**) Loading plot for the variables selected in each dataset and the 2 components. The most important variables are ordered from bottom to top. Colors indicate the group for which the median expression value is the highest for each feature: CON, blue; PQ-15, orange; PQ-30, green. (**b**) Circos plot. The plot represents the correlations greater than 0.7 between variables of different omics. Each quadrant indicates the type of features: plasma metabolites (purple), urine metabolites (red), microbes (yellow). The lines show the positive (red) and negative (blue) correlations. Abbreviations: LPC, lysophospholipid; PC, phosphatidylcholine; SM, sphingomyelin; TG, triglyceride.

The final model was able to discriminate between the different omics in Figure S8a,b, as it is observed there are some dissimilarities between animals across datasets (Figure S8b). The variables with higher impact were represented in Figure S8c. In general, the correlation structure in component 1 shows correlation between specific variables at different omics, while some plasma and urine metabolites seem to highly contribute to component 2. Focusing on component 1, the most important variables are LPC 18:2 (plasma metabolite), succinic acid (urine metabolite) and *Escherichia coli* (microbes) (Figure 7a). Focusing on the multi-omics signature selected on component 2, the most important variables are a group of TGs plasma metabolites and a group of urine metabolites known as pseudouridine, 1-methylnicotinamide and N,N-dimethylglycine (Figure 7a). Additionally, the cross-correlations between omics, and the nature of these correlations are represented in Figure 7b, thus we can observe that correlations are between plasma metabolites and some urine metabolites and microbes. The majority negative correlations are observed between pseudouridine and plasma metabolites. Additionally in plasma, LPCs and two amino acids (proline and hydroxyproline) are positively correlated with other’s omics as succinic acid and fumaric acid in urine. *Escherichia coli* and *Muribaculaceae bacterium* DSM 103,720 targets the negative and positive correlations between the other omics. 

Finally, the performance of the model was assessed indicating a BER of 0.12. This rate is almost null in the PQ-30 group, while CON presents the highest error. To complement the analysis, ROC and AUC shows that PQ-15 is the most difficult group to classify with DIABLO compared to the other groups (Figure S9). Thus, multi-omics data integrations present a high prediction power highlighting the discrimination of PQ-30 versus CON and PQ-15. 

## 4. Discussion

In this study, we explored the metabolic and gut microbiome responses to PQ administration in male Wistar rats, with a particular focus on the context of oxidative stress. We accomplished this by treating the rats with a single intraperitoneal injection of 15 and 30 mg/kg PQ, inducing oxidative stress damage [14,23]. 

Interestingly, as the PQ dosage increased, the animals showed a decrease in body weight, thus suggesting that a higher dose of PQ is related to a greater impact at a metabolic level. These results are in agreement with the existing literature about the potential detrimental effects of PQ on metabolic processes and body weight regulation [24]. The pronounced reduction in food intake in both PQ-15 and PQ-30 groups, further emphasizes the disruptive influence of PQ on dietary patterns. Such alterations in feeding behavior and body weight may be indicative of underlying systemic disturbances, potentially affecting nutrient absorption, metabolism, or energy utilization. However, it is crucial to acknowledge that while these findings provide valuable insights into the immediate impact of PQ administration, they also introduce a limitation in the current experimental model. This trend was also extended to specific tissues like the liver which is a metabolic hub and a central player under oxidative stress conditions. The observed relationship in body weight and tissue alterations justified to further study the metabolic and microbiome PQ-responses.

Due to the model validation as well as to the characterization of the oxidative stress response to PQ administration, alterations in lipid peroxidation were evident in both the liver and plasma, as observed by elevated levels of MDA, consistent with prior findings in PQ animal models [25,26]. Concurrently, ROS levels were increased in PQ-treated groups. Furthermore, the urinary levels of 8-isoprostanes, widely recognized as a gold standard for assessing oxidative damage, exhibited a substantial increase at the highest PQ dose (PQ-30), consistent with findings reported in previous research [27]. Elevated expression of mitochondrial (*SOD1*) and cytoplasmic (*SOD2*) SOD and *CAT* in the liver suggested the increment of dismutation of O_2_^•−^ to H_2_O_2_. Those results were in agreement with previous studies that indicate an increased SOD activity as a main characteristic of the oxidative model with PQ administration. To further confirm our results, SOD activity was also measured; being increased in liver, while SOD activity was not altered in plasma. 

It seems that one of the major antioxidant elements against PQ poisoning is GSH, which plays a pivotal role in the defense against oxidative stress damage. This could be suggested due to the observed effect on liver GR (increased by PQ) which catalyzes the reduction of GSSG to obtain GSH. Its major antioxidant properties are further manifested in direct scavenging of hydroxyl radicals and singlet oxygen, while it can also detoxify H_2_O_2_ and lipid peroxides simultaneously to the enzymatic action of GPx and glutathione transferases [28]. Furthermore, it could be also speculated that H_2_O_2_ decomposition is not greatly increased because GPx1 expression is not changed along with the decrease in CAT expression. These findings are in line with similar experiments with PQ administration [29,30], where the protective effect of GR was enhanced and GPx1 remained unaffected.

Although the mechanism of toxicity of PQ has not been fully described, it seems that one of the most important mechanisms of PQ damage is through the induction of inflammation [31]. This effect was also confirmed in our study due to the elevated levels of MCP-1, which is proposed as biomarkers for monitoring PQ-mediating inflammation [32]. Beyond the elevated levels of MCP-1, the PQ-treated groups also present increased excretion levels of N-acetylglycoproteins in urine, which have been proposed as biomarkers of systemic inflammation [33]. Altogether, these findings strongly support the concept that PQ toxicity is strongly associated with the activation of systemic inflammation.

**Figure 8:**
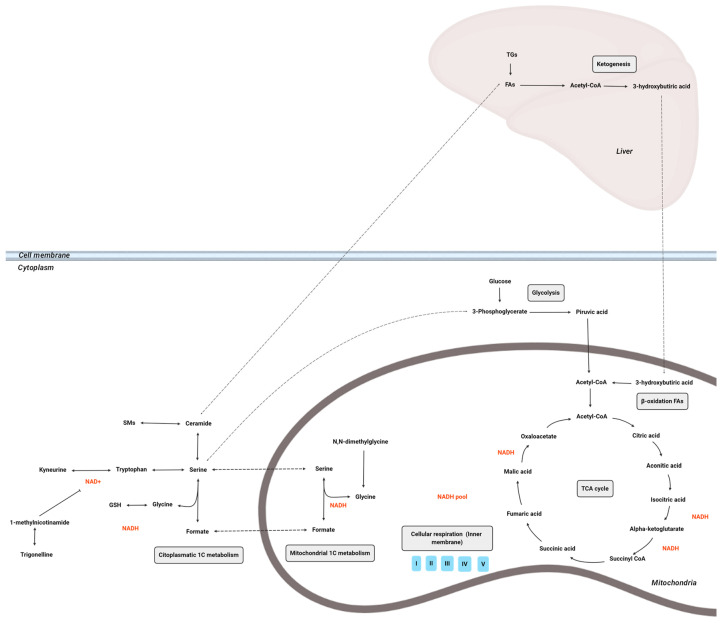
Overview of the main metabolic pathways implicated in the PQ oxidative stress model. Abbreviations: TG, triacylglycerol; FA, fatty acid; SM, sphingomyelin; GSH, glutathione; NAD, nicotinamide adenine dinucleotide; complex I, NADH:ubiquinone oxidoreductase; complex II, succinate dehydrogenase; complex III, ubiquinol:cytochrome c oxidoreductase; complex IV, cytochrome c oxidase; complex V, ATP synthase.

On the other hand, the mitochondrial stress generated by PQ administration, which has been confirmed by classical biomarkers such as MDA, SOD, CAT, GR and GPx1, was also observed at a plasmatic metabolic level. In this regard, the plasma metabolic profile of PQ-toxicity was characterized by altered metabolites associated with mitochondrial metabolism along with other related pathways, as summarized in Figure 8. In fact, it has long been recognized that energy metabolism is linked with the production of ROS. Critical metabolites allied to metabolic pathways can be affected by redox reactions [34]. Mammals produce an important part of its metabolic energy from carbohydrates, with glucose being the principal substrate for energy production (ATP), that is produced through three well-known pathways: glycolysis, tricarboxylic acid (TCA) cycle and the electron transport chain.

The TCA cycle, which constitutes an epicenter in cell metabolism, is one of the main PQ-altered pathways in the mitochondria [35]. In the current research, citric acid and aconitic acid, which are intermediates of TCA cycle, were increased in the PQ-15 group while they were decreased in the PQ-30. Thereby, our experimental approach suggests that in moderate oxidative stages (PQ-15), TCA cycle increases in parallel to ketonic bodies accumulation; while in higher altered oxidative stages (PQ-30), the TCA cycle was disrupted, but the ketone bodies continue to accumulate. The H_2_O_2_ accumulation leads to the suppression of the TCA cycle through the inhibition of enzymes implicated in the process, limiting the availability of reduced nicotinamide adenine dinucleotide (NADH) for the respiratory chain under oxidative stress. On the assumption that H_2_O_2_ levels determine their effect on the TCA cycle and taking into account the results obtained for PQ-15 and PQ-30, it is likely to suggest two states for each dose, as in agreement with previous studies [36,37]: (1) low H_2_O_2_ concentrations inactivate aconitase, the most sensitive enzyme in the TCA cycle, to H_2_O_2_ inhibition, thus glutamate fuels the TCA cycle and NADH generation is unaltered (PQ-15), (2) high H_2_O_2_ concentrations also inhibits α-ketoglutarate dehydrogenase, limiting the amount of NADH available for the respiratory chain (PQ-30). This disruption of the TCA cycle is also evident in urine metabolites of the PQ-30 group. 

A crucial metabolite linking the TCA cycle and glycolysis is acetyl-CoA, a fundamental intermediate for ATP production. In fact, acetyl-CoA is the major product of fatty acid catabolism and plays a key role in ketogenesis [38]. The main altered ketone body in our animal model was 3-hydroxybutiric acid, which functions as a stress molecule response and helps organisms to overcome stressful/pathological situations by triggering a molecular program for stress resistance such as calorie restriction and starvation [39]. In fact, prolonged fasting events cause a decrease in glucose levels, while the production of ketone bodies increases at the expense of liver β-oxidation of adipose tissue-derived fatty acids [40]. In our study, we observed that the oxidative stress response leads to a decrease in caloric intake. This could suggest a link between the PQ related oxidative stress, and changes in the eating behavior. However, the mechanism linking fasting, and PQ is still lacking. In this regard, it has been suggested that 3-hydroxybutiric acid mediates the beneficial effects of calorie restriction through its antioxidant activity, by several mechanisms: (1) it acts as a direct antioxidant [41]; (2) it inhibits mitochondrial ROS production through NADH oxidation [42]; and (3) it promotes transcriptional activity of antioxidant defenses [43]. 

During fasting, the brain relies on ketone bodies as an alternative energy source. However, this metabolic shift towards ketogenesis is linked with an impaired mitochondrial respiration dysfunction and an elevation in H_2_O_2_, which has been observed in various neurodegenerative diseases [44]. These changes in mitochondrial function and shift to ketone body utilization in the brain, have been linked to a mechanistic pathway that connects early decline in mitochondrial respiration and H_2_O_2_ production to activation of pathways that catabolize myelin lipids (myelin to SMs, SMs to ceramide, ceramide to FAs and FAs to ketone bodies) resulting in white matter degeneration [45]. These lipids act as a local source of ketone body generation via astrocyte mediated β-oxidation of fatty acids. Astrocyte derived ketone bodies can then be transported to neurons where they undergo ketolysis to generate acetyl-CoA for TCA derived ATP generation required for synaptic and cell function [45]. Hence, we suggest a similar effect in our model due to the similar pattern found at both PQ doses, where an of increase in 3-hydroxybutiric acid expression is linked to the increase in SMs.

Indeed, the generation of ROS regulates SMs pathways, specifically this pathway is reversibly activated by H_2_O_2_ and reversibly inhibited by GSH. In our experimental approach, we suggest that the SMs pathway could be up-regulated due to a hypothetical elevated amount of H_2_O_2_; in parallel to the fact that GSH could not inhibit the activation of the pathway due to the large amount of H_2_O_2_ available. In previous studies, SM signaling cascade has been connected to oxidation stress in cell cycle and apoptotic signaling [46]. The SMs are recognized as a ubiquitous signaling system that links specific cell-surface receptors and environmental stresses with the nucleus [47]. Thus, the crosstalk between the oxidative system and SM metabolism could have important implication for developing apoptosis which plays key role in NCD. In concordance, our data reflect an increase in total SMs in the PQ-treated groups being more pronounced in the PQ-30 group following a dose-dependent pattern. 

Serine, which was increased in PQ-15 and decreased in PQ-30, is a key amino acid acting as a central node linking glycolysis to GSH synthesis and one-carbon (1C) metabolic cycle, which are closely related to its antioxidant capacity [48]. 1C metabolism intermediary products regulate oxidative stress with the production of NADH and GSH, which have an intrinsic ROS scavenging capacity. In previous studies, it has been shown that serine decreased when oxidative raised, thus favoring the development of metabolic syndrome and obesity [49,50]. In line with this, our present results show decreased values of serine in the PQ-30 group while this amino acid rises its concentration in the PQ-15 group; this fact suggests that the PQ-15 animals are trying counterbalance the ROS by increasing the serine levels. In addition, an in vitro study showed that serine deficiency causes a higher response to oxidative stress and higher ROS content as it is shown in our case for PQ-30 [51].

Furthermore, elevated levels of LPC have been associated with oxidative stress through ROS generation and systemic inflammation [52]. Interestingly, the LPC levels were decreased in the PQ-induced oxidative stress groups (PQ-15 and PQ-30). LPC, which is the main component of oxLDL, originates from the cleavage of the membrane PC by phospholipase A_2_ (PLA_2_). In this sense, inactivation of PLA_2_, which could explain the low LPC levels detected in our PQ exposed rats could be attributed to the Fenton reaction as it has been previously reported [53]. Additionally, it has also been proposed that LPC-induced oxidative stress may have a dual effect depending on the amount and type of ROS, the duration of exposure and the type of cell [54]. 

In this experimental approach, the plasma metabolome provides a broader picture of the metabolic changes compared to the urine metabolome, nonetheless there are interesting metabolites that could be also discussed and linked to the plasma metabolic profile. Related to energy metabolism, the TCA cycle was decreased in group PQ-30 as it is previously described for plasma. For instance, 1-methylnicotinamide, a major urinary product of nicotinamide metabolism, was increased in animals with PQ-induced oxidative stress. Interestingly, 1-methylnicotinamide has been shown to inhibit NAD+ synthesis participating in redox homeostasis, making it a central player for energy metabolism [55]. Additionally, trigonelline, a methylated 1-methylnicotinamide, was also decreased in the PQ-30 group. 

On the other hand, regarding NAD+ synthesis, tryptophan is degraded to produce NAD+ in the kynurenine pathway that can be activated by stress and immunocytokines. Thus, the decrease in tryptophan in PQ-30 groups suggests that tryptophan was displaced to generate NAD+. Those altered metabolites reflect the importance of NADH metabolism in energy metabolism and the subsequent development of metabolic disorders [56]. In previous studies, pseudouridine has been considered a secreted urinary oxidative stress biomarker, reflecting RNA turnover since it is originated from degraded rRNA and tRNA [57]. The PQ-15 group followed the general tendency of increased pseudouridine in relation to oxidative stress [58], while the PQ-30 group presented similar values to the CON group. That fact could likely be explained due to pseudouridine accumulating in other tissues as previously reported in the case of renal failure [59]. 

For the global assessment of the toxicity of environmental contaminants on the body, the gut microbiota is an important but often overlooked factor [60]. Research on the effects of PQ on the gut microbiota has been limited, with few studies conducted on piglets [13], flies [61] and mice [12]. Our findings on the gut microbiome from rats indicate that high doses of PQ (PQ-30 group) lead to paramount changes in microbial population. We suggest that the increase in oxidative molecules in the gut microbiome results in the reduction in specific microorganisms sensitive to PQ. This phenomenon may explain the non-homogeneous decrease in microbial diversity within the gut microbiome. For instance, one of the latest studies on early PQ exposure indicated that PQ reduced gut microbiota diversity altered the structure of gut microbiota in an early-life murine model [12]. 

Our results show that the microbiota changed in the PQ-15 group, but the change was much more dramatic in the PQ-30 group. While PQ-15, at a lower dose, may not have reached a concentration threshold able to induce significant shifts in the microbiome, the PQ-30 dose could have surpassed a critical level, leading to pronounced microbiome alterations. Moreover, the gut microbiome is known for its resilience but can undergo substantial changes in response to external stimuli [62]. The intense sensitivity to PQ may indicate a dose-dependent threshold where the microbiome could suffer more pronounced shifts in response to the external stressor.

In the present study, the relative abundance of *Firmicutes* decreased, which is widely associated with obesity, as shown in the PQ-15 group. On the other hand, in the PQ-30 group the relative abundance of *Firmicutes* and *Bacteroidetes* dramatically decreased in favor of *Proteobacteria* and *Verrucomicrobia*. Those changes suggest that *Proteobacteria* and *Verrucomicrobia* have efficient mechanisms to manage oxidative stress in comparison to other phyla.

On the one hand, *Escherichia coli*, which was the main altered species in the *Proteobacteria* phylum and positively associated with isoprostanes, has several major regulators activated during oxidative stress. Those regulators are functionally conserved in a broad range of bacterial groups in *Proteobacteria* (SoxRS and RpoS), reflecting a positive selection of these regulators [63]. This could explain why *E. coli* presented a different pattern being the most resistant in the PQ-30 group. 

On the other hand, *Akkermansia muciniphila*, a mucin-degrading Gram-negative bacteria, belonging to the *Verrucomicrobia* phylum [64], is the second-most dominant species of the PQ-30 group and was positively associated with isoprostanes. High inflammation has previously been associated with a decreased abundance of *A. muciniphila* such as in cases of ulcerative colitis patients [65]. *A. muciniphila* is associated with health benefits including protection against cardiometabolic disorders such as diabetes and obesity [66,67], hence its dominance may represent an adaptive response to elevated ROS and systemic inflammation. Although *A. muciniphila* degrades mucus, its presence increases the number of goblet cells which secrete mucus, thereby strengthening the protective barrier of the intestine. In addition, *A. muciniphila* derived melatonin production has been found to scavenge ROS [68]. Orally administered *A. muciniphila* in diabetic rats has demonstrated a reduction in systemic inflammation, a decrease in gut permeability and reduction in MDA [69]. Its health benefits are suggested to influence in an indirect fashion, creating conditions for beneficial microbes and metabolites, or directly via heat-resistant surface protein, Amuc_1100 or production of short-chain fatty acids, butyrate and propionate that contribute to the gut barrier integrity [70]. 

## 5. Conclusions

Our study reveals the systemic impact of PQ administration in male Wistar rats, emphasizing its effects on body weight, oxidative stress biomarkers, metabolome and gut microbiome. Alterations in the tricarboxylic acid cycle and ketone body accumulation highlight the intricate connection between energy metabolism, redox biology, and PQ-induced oxidative stress.

Key metabolites, such as 3-hydroxybutyric acid, suggest potential links between calorie restriction and stress resistance. Changes in serine levels provide insights into the complex network of metabolic pathways affected by PQ exposure. Plasma metabolome analysis reveals significant shifts in mitochondrial metabolism, including alterations in nicotinamide metabolism and tryptophan degradation.

Exploring the gut microbiome indicates changes, with higher PQ doses affecting microbial populations and metagenomic functions. Furthermore, our results suggest that *Proteobacteria* and *Verrucomicrobia* have efficient mechanisms to manage oxidative stress in comparison to other phyla.

Overall, this study contributes to our understanding of the mechanisms underlying PQ-induced oxidative stress metabolic alterations and the connections between xenobiotic exposure, gut microbiota and host metabolism.

## Figures and Tables

**Table 1:** Detailed sequences of the oligonucleotides used in q-PCR.

Gene	Forward Primer (5′-3′)	Reverse Primer (5′-3′)	Accession Number	Size (bp)
*Cu/Zn SOD*	GGTGGTCCACGAGAAACAAG	CAATCACACCACAAGCCAAG	NM_017050.1	98
*Mn SOD*	AAGGAGCAAGGTCGCTTACA	ACACATCAATCCCCAGCAGT	NM_017051.2	94
*Catalase*	GAATGGCTATGGCTCACACA	CAAGTTTTTGATGCCCTGGT	NM_012520.2	100
*GPx1*	TGCAATCAGTTCGGACATC	CACCTCGCACTTCTCAAACA	NM_030826.4	120
*PPIA*	CCTCGAGCTGTTTGCAGACAA	AAGTCACCACCCTGGCACATG	NM_017101.1	138

**Table 2:** Taxonomic statistical analysis of bacterial species between the CON, PQ-15 and PQ-30 groups. Taxonomic data are presented as the mean of relative abundance (%). The summary of the analysis is shown including results of Kruskal–Wallis corrected by HS. * Denotes *p* < 0.1 (tendency), ** *p* < 0.05 (significantly different) and *** *p* < 0.01 (high significantly different).

			Corrected *p*-Value			Relative Abundance (%)	
Specie	CON vs. PQ-15 vs. PQ-30	PQ-15 vs. PQ-30	CON vs. PQ-30	CON vs. PQ-15	CON	PQ-15	PQ-30
*Muribaculaceae bacterium DSM 103720*	<0.01 ***	0.02 **	<0.01 ***	1.00	30.40%	34.43%	0.03%
*Akkermansia muciniphila*	<0.01 ***	0.02 **	<0.01 ***	1.00	2.30%	2.75%	19.31%
*Muribaculum intestinale*	<0.01 ***	0.02 **	<0.01 ***	1.00	18.49%	18.51%	-
*Bifidobacterium pseudolongum*	0.01 **	0.02 **	<0.01 ***	1.00	-	-	0.15%
*Anaerotruncus* sp. *G3 2012*	0.01 **	0.02 **	<0.01 ***	1.00	1.87%	1.44%	0.04%
*Escherichia coli*	0.01 **	0.02 **	<0.01 ***	1.00	10.79%	6.09%	76.00%
*Oscillibacter* sp. *1 3*	0.01 **	0.02 **	0.08 *	0.55	1.90%	3.80%	0.09%
*Lactobacillus johnsonii*	0.02 **	0.03 **	0.01 **	1.00	0.07%	0.01%	0.16%
*Bacteroides uniformis*	0.03 **	0.28	0.01 **	0.92	4.55%	2.18%	0.10%
*Ruthenibacterium lactatiformans*	0.03 **	0.02 **	0.04 **	1.00	0.23%	0.03%	0.69%
*Faecalibaculum rodentium*	0.04 **	0.05 *	0.04 **	1.00	-	-	0.04%

## Data Availability

The data presented in this study are available on request from the corresponding author. The data are not publicly available in the interest of performing more analysis for further publications together with more data.

## Supplementary Materials

The following supporting information can be downloaded at: https://www.mdpi.com/article/10.3390/antiox13010067/s1, Supplementary Figure S1: Full unedited gels for Figure 2i. Supplementary Figure S2: Venn-diagram of plasma metabolites of the PQ-induced oxidative stress model. The numbers correspond to the total number of metabolites presenting statistical differences. Supplementary Figure S3: Multivariate analysis’s summary of plasma metabolome in the PQ-induced oxidative stress model. (a) PCA scores of plasma metabolome. (b) PLS-DA scores of plasma metabolome. The Score plot is represented, and it includes the number of components, the cumulative R2X, R2Y and Q2Y. Groups (*n* = 10 animals per group): CON, blue; PQ-15, orange; PQ-30, green. Supplementary Figure S4: Venn-diagram of urine metabolites of the PQ-induced oxidative stress model. The numbers correspond to the total number of metabolites presenting statistical differences. Supplementary Figure S5: Multivariate analysis’s summary of urine metabolome in the PQ-induced oxidative stress model. (a) PCA scores of urine metabolome. (b) PLS-DA scores of urine metabolome. The Score plot is represented, and it includes the number of components, the cumulative R2X, R2Y and Q2Y. Groups (*n* = 10 animals per group): CON, blue; PQ-15, orange; PQ-30, green. Supplementary Figure S6: Summary of the virus statistical analysis in the PQ-induced oxidative stress model. (a) Beta diversity: PCA plot calculated by Aitchison distance. (b) Alpha diversity (AU): Chao1 index. (c) Relative distribution of virus phylum. (d) Relative distribution of virus species. Groups (*n* = 8 animals per group): CON, blue; PQ-15, orange; PQ-30, green. Supplementary Figure S7: Relative distribution of functions between the experimental groups (*n* = 8 animals per group) of the PQ-induced oxidative stress model. Supplementary Figure S8: Multi-omics data integration of plasma metabolome, urine metabolome and microbiome using DIABLO in the PQ-induced oxidative stress model. (a) Sample plot. The samples, which are plotted according to their scores on the 2 components for each data set, are associated showing the degree of agreement between the different data sets and the discriminative ability of each data set. Samples are coloured by group: CON; blue, PQ-15; orange and PQ-30; green. (b) Arrow plot. The samples are projected into the space spanned by the first 2 components for each data set then overlaid across data sets. The start of the arrow indicates the centroid between all data sets for a given sample and the tip of the arrow the location of the same sample in each block. Arrows further from their centroid indicate some disagreement between data sets. Samples are coloured by group (CON; blue, PQ-15; orange and PQ-3; green) and data sets are shaped (plasma metabolome: circle, urine metabolome: triangle and microbiome: cross). (c) Correlation circle plot. The plot highlights the potential associations within and between different variable types. Clusters of points indicate a strong elation between variables. Each colour and shape indicate the type of features: plasma metabolome (purple circle), urine metabolites (red triangle) and finally, microbiome (yellow cross). Supplementary Figure S9: ROC and AUC based on DIABLO performed on the PQ-induced oxidative stress model. This figure shows the ROC curve and AUC for one class versus the others for each data set (plasma metabolome, urine metabolome and microbiome) and the 2 components. The Wilcoxon test p-value is calculated to assess the differences between the predicted components from one class versus the others. ** *p* < 0.05 (significantly different) and *** *p* < 0.01 (high significantly different). Supplementary Table S1: General characteristics of the PQ-induced oxidative stress model. The results are presented as the mean ± SEM (*n* = 10 animals per group). The biometric parameters are represented as a percentage of g per kg of body weight to allow for proper comparison of parameters. The statistical comparisons among groups were conducted using 1-way ANOVA and post hoc (Tukey) test. * Denotes *p* < 0.1 (tendency), ** *p* < 0.05 (significantly different) and *** *p* < 0.01 (high significantly different) compared with control. Abbreviations: BW, body weight; MWAT, mesenteric white adipose tissue; RWAT, retroperitoneal white adipose tissue; TG, triglycerides; TC, total cholesterol; NEFAs, non-esterified fatty acids. Supplementary Table S2: Statistical analysis of plasma metabolites in the PQ-induced oxidative stress model. CON, PQ-15 and PQ-30 groups (*n* = 10 animals per group) are represented by relative abundances (AU). Relative abundances of metabolites are presented by the mean ± SEM. Plasma metabolites are shorted by VIPs. The summary of univariant and multivariate analysis is shown including *p*-value (Kruskal-Wallis test), *q*-value (correction with False discovery rate Benjamini-Hochberg test), post-Hoc test between groups if there are significant differences in Krustal-Wallis test and VIP value of PLS-DA. The statistically significant values (< 0.05) are highlighted in bold. Abbreviations: DG, diacylglycerol; ChoE, cholesterol ester; TG, triglyceride; PC, phosphatidylcholine; SM, sphingomyelin; LPC, lysophospholipid; PE, phosphatidylethanolamine. Supplementary Table S3: Statistical analysis of urine metabolites in the PQ-induced oxidative stress model. CON, PQ-15 and PQ-30 groups (*n* = 10 animals per group) are represented by relative abundances (AU). Relative abundances of metabolites are presented by the mean ± SEM. Urine metabolites are shorted by VIPs. The summary of univariant and multivariate analysis is shown including *p*-value (Kruskal-Wallis test), *q*-value (correction with False discovery rate Benjamini-Hochberg test), post-Hoc test between groups if there are significant differences in Krustal-Wallis test and VIP value of PLS-DA. The statistically significant values (<0.05) are highlighted in bold. Supplementary Table S4: Summary of bacteria phyla in the CON, PQ-15 and PQ-30 groups (*n* = 8 animals per group). The summary of univariant analysis is shown including results of Kruskal-Wallis (*p*-value), Kruskal-Wallis corrected by HS (q-value) and FC, the statistically significant values (<0.05) are highlighted in bold. Taxonomic data is presented as the mean of relative abundance (%). Supplementary Table S5: Summary of bacteria species in the CON, PQ-15 and PQ-30 groups (*n* = 8 animals per group). The summary of univariant analysis is shown including results of Kruskal-Wallis (*p*-value), Kruskal-Wallis corrected by Holm-Šídák (*q*-value) and FC, the statistically significant values (<0.05) are highlighted in bold. Taxonomic data is presented as the mean of relative abundance (%). Supplementary Table S6: Summary of virus species in the CON, PQ-15 and PQ-30 groups (*n* = 8 animals per group). The summary of univariant analysis is shown including results of Kruskal-Wallis (*p*-value), Kruskal-Wallis corrected by Holm-Šídák (*q*-value) and FC, the statistically significant values (<0.05) are highlighted in bold. Taxonomic data is presented as the mean of relative abundance (%). Supplementary Table S7: Statistically significant differences in functions between the experimental groups of the PQ-induced oxidative stress model. In this table, the most abundant functions are represented in CON, PQ-15 and PQ-30 groups (*n* = 8 animals per group). The summary of analysis is shown including results of Kruskal-Wallis corrected by HS (*q*-value) and % relative of each function, the statistically significant values (<0.05) are highlighted in bold. Supplementary Table S8: Multi-omics data association with isoprostanes (gold standard biomarker). The multi-omics data was given as input to MaAslin2 comprehensive R package (Multivariate Association with Linear Models 2, v.1.8.0—Bioconductor) alongside isoprostanes levels. Results were considered significant if they had a q-value smaller than 0.25.
