# Supplementary material for: Exploring Metabolic and Gut Microbiome Responses to Paraquat Administration in Male Wistar Rats: Implications for Oxidative Stress
Source: Antioxidants (Basel). 2024 Jan 1;13(1):67. doi: 10.3390/antiox13010067 (PMC10812659; doi:10.3390/antiox13010067)
Supplement: Supplementary file 1 [file antioxidants-13-00067-s001.zip › antioxidants-2773870-supplementary.pdf]

Supplementary Table S1. General characteristics of the PQ-induced oxidative stress model. The results are presented as the mean ± SEM (n = 10 animals per group). The biometric parameters are represented as a percentage of g per kg of body weight to allow for proper comparison of parameters. The statistical comparisons among groups were conducted using 1-way ANOVA and post hoc (Tukey) test. * Denotes $p < 0.1$ (tendency), ** $p < 0.05$ (significantly different) and *** $p < 0.01$ (high significantly different) compared with control. Abbreviations: BW, body weight; MWAT, mesenteric white adipose tissue; RWAT, retroperitoneal white adipose tissue; TG, triglycerides; TC, total cholesterol; NEFAs, non-esterified fatty acids.

	CON	PQ-15	PQ-30	*p*-value	CON vs PQ-15	CON vs PQ-30	PQ-15 vs 30
Inicial body weight (g)	286.21 ± 4.24	286.37 ± 3.24	300.85 ± 8.28	0.11	0.94	0.7	0.33
Final body weight (g)	293.77 ± 3.90	271.59 ± 5.54	263.35 ± 7.37	<0.01***	0.03**	<0.01***	0.58
Food intake (g)	21.19 ± 0.77	11.47 ± 1.44	0.65 ± 0.20	<0.01***	<0.01***	<0.01***	<0.01***
MWAT weight (%)	0.86 ± 0.02	0.87 ± 0.5	0.71 ± 0.06	0.05*	0.99	0.09*	0.76
RWAT weight (%)	1.09 ± 0.5	1.03 ± 0.09	0.88 ± 0.13	0.36	0.91	0.31	0.55
Muscle weight (%)	0.63 ± 0.01	0.60 ± 0.01	0.56 ± 0.01	<0.01***	0.17	<0.01***	<0.01***
Liver weight (%)	3.61 ± 0.04	3.26 ± 0.10	2.55 ± 0.06	<0.01***	<0.01***	<0.01***	<0.01***
Cecum weight (%)	1.55 ± 0.09	1.37 ± 0.07	0.97 ± 0.11	<0.01***	0.37	<0.01***	0.13
Plasma Glucose (mM)	114.39 ± 1.63	108.47 ± 3.74	119.10 ± 5.33	0.17	0.53	0.67	0.14
Plasma TG (mM)	81.05 ± 7.96	55.29 ± 12.11	57.12 ± 5.70	0.09*	0.13	0.16	0.98
Plasma TC (mM)	67.44 ± 3.09	76.62 ± 5.55	94.33 ± 4.83	<0.01***	0.35	<0.01***	0.03**
Plasma NEFAs (mM)	0.70 ± 0.04	0.63 ± 0.03	0.68 ± 0.05	0.39	0.39	0.95	0.56

Full unedited gels for Figure 2i

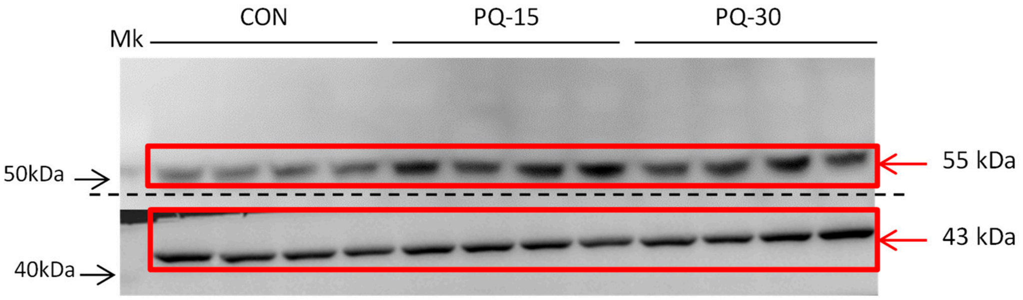

Upper membrane: anti- glutathione reductase
Lower membrane: : anti-β-actin

Supplementary Figure S1. Full unedited gels for Figure 2i.

Supplementary Table S2. Statistical analysis of plasma metabolites in the PQ-induced oxidative stress model. CON, PQ-15 and PQ-30 groups (*n* = 10 animals per group) are represented by relative abundances (AU). Relative abundances of metabolites are presented by the mean ± SEM. Plasma metabolites are shorted by VIPs. The summary of univariant and multivariate analysis is shown including *p*-value (Kruskal-Wallis test), *q*-value (correction with False discovery rate Benjamini-Hochberg test), post-Hoc test between groups if there are significant differences in Krustal-Wallis test and VIP value of PLS-DA. The statistically significant values (< 0.05) are highlighted in bold. Abbreviations: DG, diacylglycerol; ChoE, cholesterol ester; TG, triglyceride; PC, phosphatidylcholine; SM, sphingomyelin; LPC, lysophospholipid; PE, phosphatidylethanolamine.

Metabolite	CON	PQ-15	PQ-30	*p*-value	*q*-value	CON vs PQ-15	CON vs PQ-30	PQ-15 vs PQ-30	VIP
Aconitic acid	0.005 ± 0.0002	0.011 ± 0.0011	0.002 ± 0.0002	**<0.01**	**<0.01**	**<0.01**	**<0.01**	**<0.01**	1.7
Citric acid	4.41 ± 0.11	5.09 ± 0.11	2.75 ± 0.14	**<0.01**	**<0.01**	**<0.01**	**<0.01**	**<0.01**	1.6
SM 36:1	1.39 ± 0.05	2.19 ± 0.28	5.28 ± 0.34	**<0.01**	**<0.01**	**0.02**	**<0.01**	**0.02**	1.6
3-hydroxybutiric acid	1.61 ± 0.06	2.54 ± 0.29	4.58 ± 0.19	**<0.01**	**<0.01**	**0.02**	**<0.01**	**0.02**	1.6
LPC 20:0	0.31 ± 0.01	0.34 ± 0.02	0.14 ± 0.01	**<0.01**	**<0.01**	0.39	**<0.01**	**<0.01**	1.6
LPC 15:0	0.96 ± 0.02	0.81 ± 0.06	0.45 ± 0.02	**<0.01**	**<0.01**	**0.02**	**<0.01**	**0.02**	1.6
SM 36:2	0.53 ± 0.02	0.74 ± 0.07	1.41 ± 0.09	**<0.01**	**<0.01**	**0.02**	**<0.01**	**0.02**	1.6
ChoE (22:6)	5.09 ± 0.41	5.16 ± 0.58	13.07 ± 0.66	**<0.01**	**<0.01**	0.83	**<0.01**	**<0.01**	1.6
SM 34:1	22.72 ± 0.67	31.58 ± 2.67	46.87 ± 1.81	**<0.01**	**<0.01**	**0.01**	**<0.01**	**0.03**	1.6
Serine	0.5 ± 0.04	0.74 ± 0.04	0.16 ± 0.03	**<0.01**	**<0.01**	**0.02**	**<0.01**	**<0.01**	1.5
SM 34:2	1.93 ± 0.06	2.42 ± 0.14	3.36 ± 0.13	**<0.01**	**<0.01**	**0.02**	**<0.01**	**0.03**	1.5
SM 38:1	0.56 ± 0.02	0.88 ± 0.11	1.49 ± 0.09	**<0.01**	**<0.01**	**0.01**	**<0.01**	0.06	1.5
LPC 18:1	17.22 ± 0.7	11.85 ± 0.83	8.15 ± 0.35	**<0.01**	**<0.01**	**0.01**	**<0.01**	**0.03**	1.5
LPC 18:2	52.7 ± 1.09	46.39 ± 4.11	24.49 ± 1.2	**<0.01**	**<0.01**	0.08	**<0.01**	**0.01**	1.5
Cholesterol	0.11 ± 0.01	0.16 ± 0	0.08 ± 0.01	**<0.01**	**<0.01**	**<0.01**	**0.02**	**<0.01**	1.5
Urea	0.77 ± 0.02	0.8 ± 0.03	1.11 ± 0.05	**<0.01**	**<0.01**	0.76	**<0.01**	**<0.01**	1.4
PC 32:2	0.36 ± 0.02	0.21 ± 0.04	0.12 ± 0.01	**<0.01**	**<0.01**	**<0.01**	**<0.01**	0.09	1.4
SM 35:1	0.23 ± 0.01	0.39 ± 0.04	0.42 ± 0.03	**<0.01**	**<0.01**	**<0.01**	**<0.01**	0.33	1.4
DG 34:3	0.79 ± 0.06	0.36 ± 0.03	0.41 ± 0.04	**<0.01**	**<0.01**	**<0.01**	**<0.01**	0.54	1.4
Fumaric acid	0.9 ± 0.04	0.6 ± 0.04	1.12 ± 0.08	**<0.01**	**<0.01**	**<0.01**	0.07	**<0.01**	1.4
PC 33:1	0.13 ± 0.01	0.08 ± 0.01	0.06 ± 0	**<0.01**	**<0.01**	**<0.01**	**<0.01**	0.25	1.3
ChoE (20:4)	103.57 ± 11.17	71 ± 3.79	171.05 ± 4.16	**<0.01**	**<0.01**	0.37	**<0.01**	**<0.01**	1.3

PC 38:2	0.28 ± 0.01	0.25 ± 0.02	0.15 ± 0.01	**<0.01**	**<0.01**	0.13	**<0.01**	**0.01**	1.3
ChoE (16:1)	0.59 ± 0.06	0.27 ± 0.04	0.15 ± 0.01	**<0.01**	**<0.01**	**<0.01**	**<0.01**	0.06	1.3
SM 42:3	6.6 ± 0.23	8.87 ± 0.83	11.52 ± 0.86	**<0.01**	**<0.01**	**0.02**	**<0.01**	0.06	1.3
LPC 16:0	80.8 ± 1.99	83.7 ± 3.85	61.2 ± 1.08	**<0.01**	**<0.01**	0.67	**<0.01**	**<0.01**	1.3
Fructose	2.17 ± 0.17	1.34 ± 0.05	2.76 ± 0.17	**<0.01**	**<0.01**	**<0.01**	0.05	**<0.01**	1.3
SM 33:1	0.38 ± 0.01	0.47 ± 0.03	0.54 ± 0.03	**<0.01**	**<0.01**	**0.01**	**<0.01**	0.14	1.3
LPC 16:1 e	0.13 ± 0	0.16 ± 0.01	0.17 ± 0.01	**<0.01**	**<0.01**	**<0.01**	**<0.01**	0.44	1.2
PC 38:3	1.45 ± 0.11	0.76 ± 0.18	0.68 ± 0.06	**<0.01**	**<0.01**	**<0.01**	**<0.01**	0.91	1.2
TG 46:0	0.35 ± 0.06	0.05 ± 0.02	0.44 ± 0.03	**<0.01**	**<0.01**	**<0.01**	0.49	**<0.01**	1.2
ChoE (22:5)	1.46 ± 0.12	1.1 ± 0.1	2.13 ± 0.15	**<0.01**	**<0.01**	0.06	**0.01**	**<0.01**	1.2
ChoE (16:0)	1.18 ± 0.06	1.6 ± 0.09	1.04 ± 0.12	**<0.01**	**<0.01**	**<0.01**	0.44	**<0.01**	1.2
DG 34:2	2.62 ± 0.11	2.01 ± 0.07	2.52 ± 0.09	**<0.01**	**<0.01**	**<0.01**	0.88	**<0.01**	1.2
PC 32:1	0.96 ± 0.12	0.4 ± 0.08	0.35 ± 0.04	**<0.01**	**<0.01**	**<0.01**	**<0.01**	0.72	1.2
SM 40:1	5.74 ± 0.23	7.6 ± 0.7	8.33 ± 0.44	**<0.01**	**<0.01**	**0.02**	**<0.01**	0.19	1.1
SM 40:2	0.93 ± 0.03	1.2 ± 0.09	1.34 ± 0.1	**<0.01**	**<0.01**	**<0.01**	**<0.01**	0.35	1.1
DG 34:1	1.45 ± 0.06	1.07 ± 0.07	1.23 ± 0.06	**<0.01**	**<0.01**	**<0.01**	0.10	0.10	1.1
Oleic acid	1.38 ± 0.04	1.6 ± 0.08	1.25 ± 0.03	**<0.01**	**<0.01**	0.06	**0.03**	**<0.01**	1.1
TG 46:1	0.2 ± 0.04	0.03 ± 0.02	0.18 ± 0.01	**<0.01**	**<0.01**	**<0.01**	0.89	**<0.01**	1.1
DG 36:2	1.6 ± 0.09	1.16 ± 0.06	1.22 ± 0.05	**<0.01**	**<0.01**	**<0.01**	**0.01**	0.66	1.1
TG 46:2	0.13 ± 0.02	0.02 ± 0.01	0.11 ± 0.01	**<0.01**	**<0.01**	**<0.01**	0.90	**<0.01**	1.1
PC 38:4	26.42 ± 0.86	33.53 ± 3.19	35.9 ± 1.41	**<0.01**	**<0.01**	0.06	**<0.01**	0.13	1.1
ChoE (18:1)	3.2 ± 0.13	3.84 ± 0.21	2.81 ± 0.16	**<0.01**	**<0.01**	**0.04**	0.08	**<0.01**	1.0
Glycolic acid	12.88 ± 0.44	13.1 ± 0.58	28.56 ± 5.95	**<0.01**	**<0.01**	0.89	**<0.01**	**<0.01**	1.0
Alpha-ketoglutarate	2.46 ± 0.13	1.85 ± 0.11	1.9 ± 0.1	**<0.01**	**<0.01**	**<0.01**	**0.01**	0.66	1.0
Glucose-6-phosphate	0.6 ± 0.05	0.77 ± 0.07	0.38 ± 0.06	**<0.01**	**<0.01**	0.10	**0.02**	**<0.01**	1.0
TG 50:4	1.99 ± 0.3	0.74 ± 0.41	0.4 ± 0.09	**<0.01**	**<0.01**	**<0.01**	**<0.01**	0.89	1.0
SM 39:1	0.22 ± 0.01	0.28 ± 0.04	0.36 ± 0.03	**<0.01**	**<0.01**	0.09	**<0.01**	0.09	1.0
TG 52:6	1.29 ± 0.17	0.56 ± 0.26	0.34 ± 0.06	**<0.01**	**<0.01**	**<0.01**	**<0.01**	0.89	1.0

PC 40:5	0.83 ± 0.04	0.81 ± 0.08	1.12 ± 0.05	**<0.01**	**<0.01**	0.64	**<0.01**	**<0.01**	1.0
ChoE (22:4)	12.04 ± 0.8	11.08 ± 1.02	17.31 ± 1.43	**<0.01**	0.01	0.60	**0.01**	**0.01**	1.0
TG 48:3	0.39 ± 0.07	0.09 ± 0.06	0.13 ± 0.02	**<0.01**	**<0.01**	**<0.01**	0.06	0.08	1.0
SM 32:1	0.25 ± 0.01	0.22 ± 0.02	0.32 ± 0.02	**<0.01**	0.01	0.26	**0.02**	**0.01**	1.0
ChoE (18:0)	0.1 ± 0.01	0.16 ± 0.01	0.14 ± 0.02	**<0.01**	**<0.01**	**<0.01**	0.09	0.21	1.0
PC 30:0	0.07 ± 0	0.05 ± 0.01	0.05 ± 0.01	**<0.01**	**<0.01**	**0.01**	**0.01**	0.88	1.0
SM 41:2	1.39 ± 0.04	1.74 ± 0.11	1.79 ± 0.15	**<0.01**	**<0.01**	**0.01**	**0.01**	0.97	1.0
Glyceric acid	1.1 ± 0.08	0.84 ± 0.06	0.72 ± 0.06	**<0.01**	**<0.01**	0.06	**<0.01**	0.28	1.0
TG 54:7	6.07 ± 0.67	3.3 ± 1.26	2.19 ± 0.36	**<0.01**	**<0.01**	**<0.01**	**<0.01**	0.77	1.0
PC 32:0	0.9 ± 0.03	1.03 ± 0.08	1.16 ± 0.05	**<0.01**	**<0.01**	0.22	**<0.01**	0.13	1.0
TG 50:3	8.43 ± 1.46	2.71 ± 1.61	2.55 ± 0.71	**<0.01**	**<0.01**	**<0.01**	**0.01**	0.41	0.9
LPC 18:0 e	0.08 ± 0	0.09 ± 0	0.08 ± 0.01	**<0.01**	**0.01**	**0.02**	0.41	**0.01**	0.9
SM 42:2	22.62 ± 0.86	28.47 ± 2.98	30.21 ± 1.78	**<0.01**	**<0.01**	0.11	**<0.01**	0.24	0.9
PC 34:0	0.4 ± 0.02	0.44 ± 0.03	0.52 ± 0.02	**<0.01**	**<0.01**	0.29	**<0.01**	0.05	0.9
TG 48:2	1.26 ± 0.25	0.34 ± 0.21	0.37 ± 0.06	**<0.01**	**<0.01**	**<0.01**	**0.03**	0.13	0.9
TG 48:1	1.57 ± 0.3	0.41 ± 0.2	0.71 ± 0.11	**<0.01**	**<0.01**	**<0.01**	0.09	0.06	0.9
LPC 16:0 e	0.4 ± 0.01	0.47 ± 0.02	0.43 ± 0.03	**0.02**	**0.02**	**0.01**	0.24	0.23	0.9
TG 54:4	14.67 ± 1.6	8.1 ± 3.06	6.04 ± 0.91	**<0.01**	**<0.01**	**<0.01**	**<0.01**	0.97	0.9
PC 35:2	0.56 ± 0.02	0.52 ± 0.05	0.4 ± 0.02	**<0.01**	**<0.01**	0.15	**<0.01**	**0.04**	0.9
ChoE (20:2)	2.46 ± 0.16	1.75 ± 0.26	1.69 ± 0.16	**<0.01**	**<0.01**	**0.02**	**0.02**	0.88	0.9
Malic acid	0.44 ± 0.02	0.33 ± 0.02	0.42 ± 0.03	**0.01**	**0.02**	**0.02**	0.89	**0.03**	0.9
SM 41:1	6.17 ± 0.21	7.73 ± 0.61	7.43 ± 0.58	**0.02**	**0.03**	0.05	0.06	0.76	0.9
TG 48:0	0.97 ± 0.1	0.52 ± 0.15	0.79 ± 0.06	**0.02**	**0.02**	**0.01**	0.39	0.13	0.9
TG 52:5	9.05 ± 1.17	4.73 ± 1.96	3.68 ± 0.73	**<0.01**	**<0.01**	**0.01**	**0.01**	0.80	0.9
ChoE (18:3)	2.39 ± 0.21	1.82 ± 0.14	1.53 ± 0.1	**<0.01**	**<0.01**	0.06	**<0.01**	0.24	0.9
Histidine	0.09 ± 0.01	0.16 ± 0.02	0.08 ± 0.03	**<0.01**	**<0.01**	**0.02**	0.26	**<0.01**	0.9
PC 34:1	6.84 ± 0.45	4.93 ± 0.55	6.71 ± 0.48	**0.02**	**0.02**	**0.02**	0.91	**0.02**	0.9
TG 51:2	0.81 ± 0.11	0.41 ± 0.17	0.35 ± 0.06	**<0.01**	**<0.01**	**<0.01**	**0.01**	0.62	0.9

2-hydroxyglutaric	0.76 ± 0.05	0.73 ± 0.06	1.22 ± 0.22	**0.04**	0.05	0.77	**0.04**	**0.04**	0.9
TG 54:3	4.82 ± 0.51	3.48 ± 1.18	1.67 ± 0.23	**<0.01**	**<0.01**	**0.03**	**<0.01**	0.17	0.8
SM 42:1	23.01 ± 0.85	27.7 ± 1.9	25.74 ± 1.4	0.06	0.07	-	-	-	0.8
SM 43:1	1.79 ± 0.08	2.16 ± 0.15	1.8 ± 0.1	0.07	0.08	-	-	-	0.8
TG 50:2	14.57 ± 2.28	6.37 ± 2.89	7.49 ± 1.7	**<0.01**	**<0.01**	**<0.01**	0.08	0.19	0.8
PC 40:4	0.29 ± 0.02	0.27 ± 0.04	0.38 ± 0.02	**0.01**	**0.01**	0.25	**0.03**	**0.01**	0.8
PC 33:0	0.04 ± 0	0.05 ± 0.01	0.03 ± 0	0.05	0.06	-	-	-	0.8
LPC 18:0	48.71 ± 1.03	55.14 ± 3.51	49.58 ± 1.05	0.31	0.33	-	-	-	0.8
Pyruvic acid	30.87 ± 2.02	23.77 ± 1.8	31.08 ± 1.56	**0.02**	**0.02**	**0.02**	0.84	**0.02**	0.8
TG 50:1	3.83 ± 0.55	1.89 ± 0.84	2.07 ± 0.48	**<0.01**	**<0.01**	**<0.01**	0.07	0.25	0.8
TG 54:6	19.84 ± 1.92	12.89 ± 3.24	22.01 ± 3.29	0.05	0.06	-	-	-	0.7
TG 52:2	15.48 ± 2.48	7.62 ± 3.91	7.4 ± 1.85	**<0.01**	**<0.01**	**<0.01**	0.05	0.30	0.7
TG 54:2	2.05 ± 0.3	2.1 ± 0.72	0.49 ± 0.07	**<0.01**	**<0.01**	0.60	**<0.01**	**<0.01**	0.7
Threonic acid	1.86 ± 0.16	2.1 ± 0.12	1.43 ± 0.13	**0.02**	**0.03**	0.15	0.12	**0.02**	0.7
TG 52:3	54.19 ± 6.72	29.27 ± 12.27	36.46 ± 8.08	**0.02**	**0.03**	**0.02**	0.22	0.22	0.7
Beta-alanine	0.23 ± 0.07	0.11 ± 0.01	1.02 ± 0.54	**<0.01**	**<0.01**	0.12	**0.02**	**<0.01**	0.7
Phenylalanine	1.77 ± 0.14	2.33 ± 0.09	3.74 ± 1.31	**0.01**	**0.02**	**0.01**	0.17	0.17	0.6
Isoleucine	0.95 ± 0.5	0.8 ± 0.14	14.07 ± 9.56	**<0.01**	**<0.01**	**0.02**	**<0.01**	0.41	0.6
Valine	1.91 ± 0.83	1.56 ± 0.1	20.64 ± 13.75	**<0.01**	**<0.01**	**0.01**	**0.01**	0.76	0.6
PC 31:0	0.06 ± 0	0.06 ± 0	0.05 ± 0	0.05	0.06	-	-	-	0.6
Leucine	0.33 ± 0.18	0.28 ± 0.04	4.39 ± 2.99	**<0.01**	**<0.01**	**0.01**	**0.01**	0.83	0.6
Glycerol	2.87 ± 0.16	2.53 ± 0.14	2.47 ± 0.12	0.16	0.17	**-**	**-**	-	0.6
Aspartic acid	1.19 ± 0.4	1.14 ± 0.08	4.7 ± 2.76	**0.01**	**0.01**	**0.03**	0.22	**0.01**	0.6
Glutamine	0.31 ± 0.18	0.15 ± 0.01	1.63 ± 1.11	0.08	0.09	-	-	-	0.5
Tyrosine	1.08 ± 0.32	0.76 ± 0.05	3.21 ± 1.81	0.45	0.47	-	-	-	0.5
ChoE (18:2)	29.79 ± 1.56	26 ± 1.42	28.56 ± 1.48	0.30	0.32	-	-	-	0.5
Methionine	0.22 ± 0.05	0.23 ± 0.01	0.72 ± 0.43	**<0.01**	**0.01**	**0.03**	0.13	**<0.01**	0.5
Glycine	6.48 ± 1.36	8.09 ± 0.27	19.98 ± 11.45	**0.01**	**0.01**	**0.01**	0.51	**0.01**	0.5

Tryptophan	2.13 ± 0.46	2.51 ± 0.17	6.45 ± 3.69	**0.02**	**0.02**	**0.03**	0.44	**0.02**	0.5
TG 50:0	0.22 ± 0.04	0.15 ± 0.07	0.25 ± 0.02	**0.04**	0.05	0.06	0.47	0.05	0.5
TG 52:1	0.8 ± 0.08	0.55 ± 0.24	0.58 ± 0.11	**0.04**	0.05	0.05	0.15	0.52	0.5
Ribose	30.94 ± 3.05	26.44 ± 3.09	38.01 ± 6.78	0.44	0.46				0.5
Lactic acid	4.52 ± 0.13	4.61 ± 0.15	4.91 ± 0.21	0.32	0.34				0.5
Ornithine	6.18 ± 1.64	6.41 ± 0.32	17.84 ± 10.54	**<0.01**	**<0.01**	**0.03**	0.10	**<0.01**	0.5
Alanine	1.42 ± 0.32	1.4 ± 0.08	3.43 ± 1.92	**0.01**	**0.02**	0.08	0.08	**0.01**	0.5
Asparagine	0.17 ± 0.03	0.31 ± 0.02	0.43 ± 0.27	**<0.01**	**<0.01**	**0.02**	0.17	**<0.01**	0.5
DG 36:4	3.79 ± 0.15	3.52 ± 0.17	3.87 ± 0.05	0.27	0.30				0.5
ChoE (17:1)	0.03 ± 0.01	0.02 ± 0.01	0.04 ± 0.02	0.42	0.43				0.4
Lysine	0.49 ± 0.13	0.53 ± 0.05	1.05 ± 0.51	0.13	0.14				0.4
Threonine	2.31 ± 0.24	2.93 ± 0.12	4.22 ± 2.13	**<0.01**	**0.01**	0.07	0.07	**<0.01**	0.4
ChoE (17:0)	0.07 ± 0.03	0.03 ± 0	0.06 ± 0.02	0.88	0.88				0.4
PC 36:4	25.43 ± 0.81	27.26 ± 2.13	26.93 ± 1.04	0.62	0.63				0.4
Hydroxyproline	3.82 ± 1.27	2.9 ± 0.23	6.8 ± 4.06	**0.01**	**0.02**	0.43	**0.02**	**0.02**	0.3
Glutamic acid	0.16 ± 0.03	0.24 ± 0.01	0.23 ± 0.13	**<0.01**	**<0.01**	**0.01**	0.09	**<0.01**	0.3
Proline	0.65 ± 0.06	0.83 ± 0.07	0.67 ± 0.34	**0.01**	**0.01**	0.16	**0.04**	**0.01**	0.3
Fructose-6-phosphate	0.47 ± 0.05	0.48 ± 0.05	0.41 ± 0.06	0.40	0.42				0.3
Alpha-tocopherol	0.55 ± 0.06	0.63 ± 0.06	0.54 ± 0.14	0.17	0.18				0.3
Glucose	0.22 ± 0.01	0.24 ± 0.01	0.22 ± 0.05	**0.04**	0.05	0.14	0.22	**0.03**	0.2
PC 36:2	18.99 ± 0.64	18.62 ± 2.1	17.96 ± 0.65	0.37	0.39				0.2
Succinic acid	0.49 ± 0.01	0.49 ± 0.02	0.48 ± 0.03	0.78	0.78				0.2

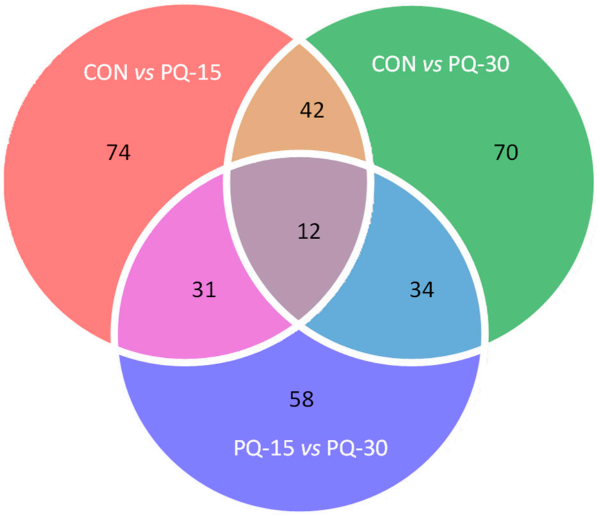

Supplementary Figure S2. Venn-diagram of plasma metabolites of the PQ-induced oxidative stress model. The numbers correspond to the total number of metabolites presenting statistical differences.

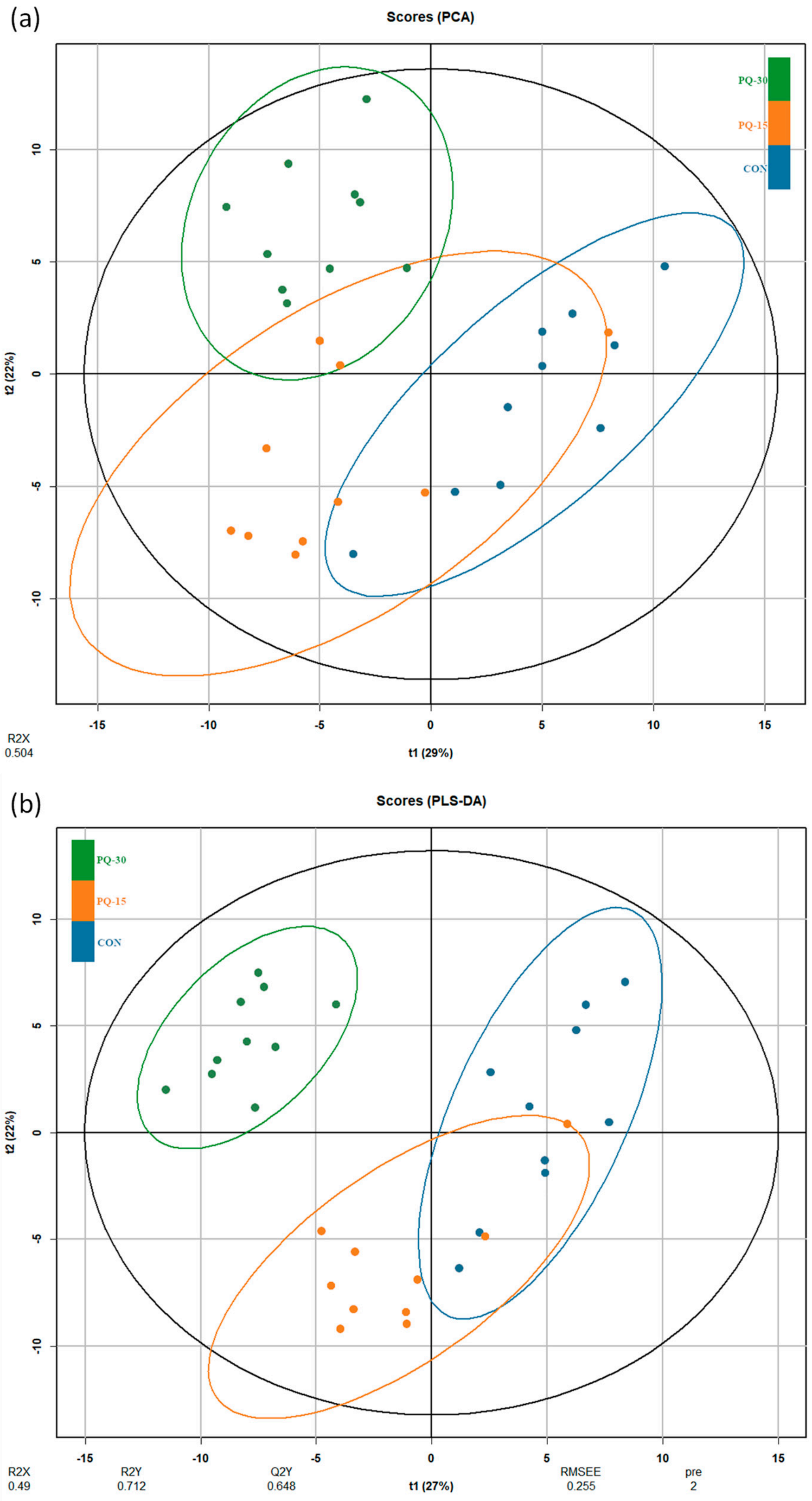

Supplementary Figure S3. Multivariate analysis's summary of plasma metabolome in the PQ-induced oxidative stress model. (a) PCA scores of plasma metabolome. (b) PLS-DA scores of plasma metabolome. The Score plot is represented, and it includes the number of components, the cumulative R2X, R2Y and Q2Y. Groups (*n* = 10 animals per group): CON, blue; PQ-15, orange; PQ-30, green.

Supplementary Table S3. Statistical analysis of urine metabolites in the PQ-induced oxidative stress model. CON, PQ-15 and PQ-30 groups (*n* = 10 animals per group) are represented by relative abundances (AU). Relative abundances of metabolites are presented by the mean ± SEM. Urine metabolites are shorted by VIPs. The summary of univariant and multivariate analysis is shown including *p*-value (Kruskal-Wallis test), *q*-value (correction with False discovery rate Benjamini-Hochberg test), post-Hoc test between groups if there are significant differences in Krustal-Wallis test and VIP value of PLS-DA. The statistically significant values (< 0.05) are highlighted in bold.

Metabolite	CON	PQ-15	PQ-30	*p*-value	*q*-value	CON vs PQ-15	CON vs PQ-30	PQ-15 vs PQ-30	VIP
Hippurate	160.93 ± 8.42	166.66 ± 6.1	-	**<0.01**	**<0.01**	0.79	**<0.01**	**<0.01**	1.52
Trigonelline	2.59 ± 0.13	2.48 ± 0.04	0.04 ± 0.04	**<0.01**	**<0.01**	0.24	**<0.01**	**<0.01**	1.44
Fumaric acid	6.62 ± 0.42	5.44 ± 0.75	0.59 ± 0.13	**<0.01**	**<0.01**	0.15	**<0.01**	**<0.01**	1.39
Succinic acid	53 ± 2.82	51.42 ± 2.07	18.94 ± 1.86	**<0.01**	**<0.01**	0.80	**<0.01**	**<0.01**	1.34
Creatinine	228.98 ± 8.09	290.18 ± 9.37	319.77 ± 16.44	**<0.01**	**<0.01**	**<0.01**	**<0.01**	0.46	1.25
Pseudouridine	12 ± 0.47	14.98 ± 0.43	12.11 ± 0.49	**<0.01**	**<0.01**	**<0.01**	0.94	**<0.01**	1.18
1-Mehtylnicotinamide	0.09 ± 0.02	0.25 ± 0.04	0.21 ± 0.03	**<0.01**	**<0.01**	**<0.01**	**0.01**	0.66	1.14
Methylamine	5.87 ± 0.33	6.24 ± 0.4	3.28 ± 0.4	**<0.01**	**<0.01**	0.93	**<0.01**	**<0.01**	1.05
Tryptophan	7.46 ± 0.47	6.67 ± 0.54	4.41 ± 0.26	**<0.01**	**<0.01**	0.57	**<0.01**	**0.01**	0.97
Citric acid	405.1 ± 17.52	375.34 ± 42.58	29.56 ± 9.7	**<0.01**	**<0.01**	0.67	**<0.01**	**<0.01**	0.92
Alanine	6.44 ± 0.32	7.46 ± 0.47	9.36 ± 0.68	**<0.01**	**<0.01**	0.11	**<0.01**	0.11	0.91
Glycine	12.06 ± 0.74	12.7 ± 0.87	17.19 ± 0.75	**<0.01**	**<0.01**	0.50	**<0.01**	**0.01**	0.90
Lactate	13.52 ± 0.77	15.94 ± 0.84	17.76 ± 0.88	**<0.01**	**0.01**	0.12	**<0.01**	0.19	0.87
Dimethylsulfone	15.41 ± 0.54	21.67 ± 2.94	17.27 ± 1.73	0.20	0.21	-	-	-	0.81
N-Acetylglycoproteins	157.07 ± 5.95	178.33 ± 6.24	171.61 ± 5.94	**0.01**	**0.01**	**0.01**	**0.04**	0.57	0.80
Formate	2.27 ± 0.21	18.35 ± 11.88	4.36 ± 0.76	0.09	0.10	-	-	-	0.79
N,N-Dimethylglycine	11.18 ± 1.16	7.75 ± 1.2	17.49 ± 3.34	**0.03**	**0.04**	0.12	0.20	**0.03**	0.72
Taurine	201.52 ± 22.06	236.57 ± 23.81	278.47 ± 28.9	0.08	0.10	-	-	-	0.64
Glucose	7.63 ± 1.63	8.57 ± 0.73	6.11 ± 0.83	0.08	0.10	-	-	-	0.50
Pyruvic acid	6.88 ± 0.41	6.93 ± 0.3	5.91 ± 0.33	0.10	0.11	-	-	-	0.43
Acetate	5.62 ± 0.37	6.05 ± 0.51	5.51 ± 0.3	0.80	0.80	-	-	-	0.30

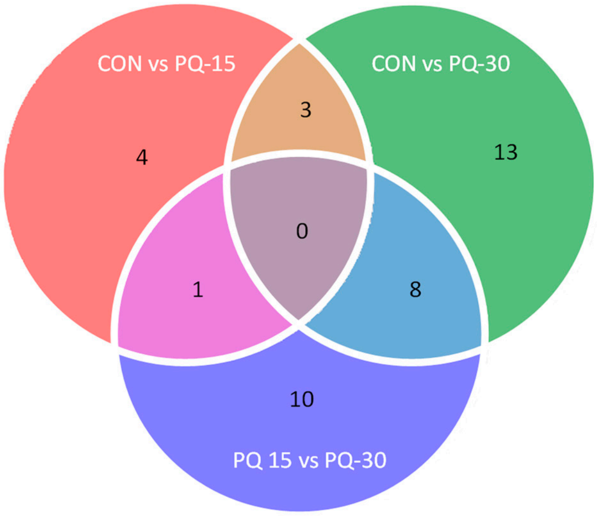

Supplementary Figure S4. Venn-diagram of urine metabolites of the PQ-induced oxidative stress model. The numbers correspond to the total number of metabolites presenting statistical differences.

(a)

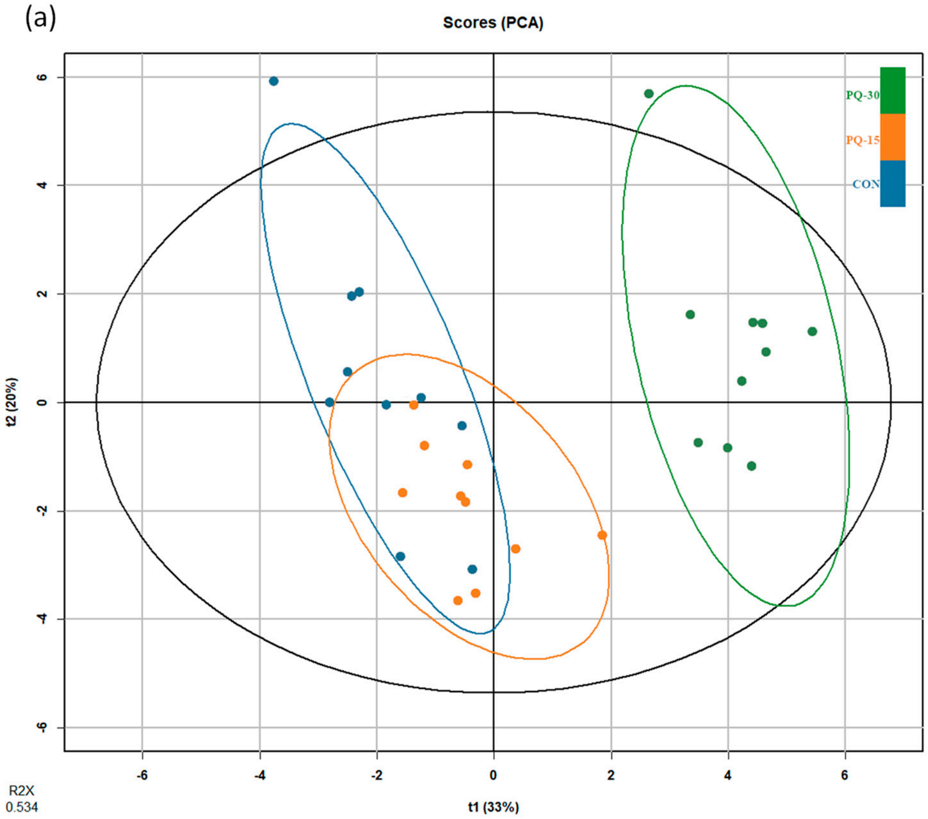

(b)

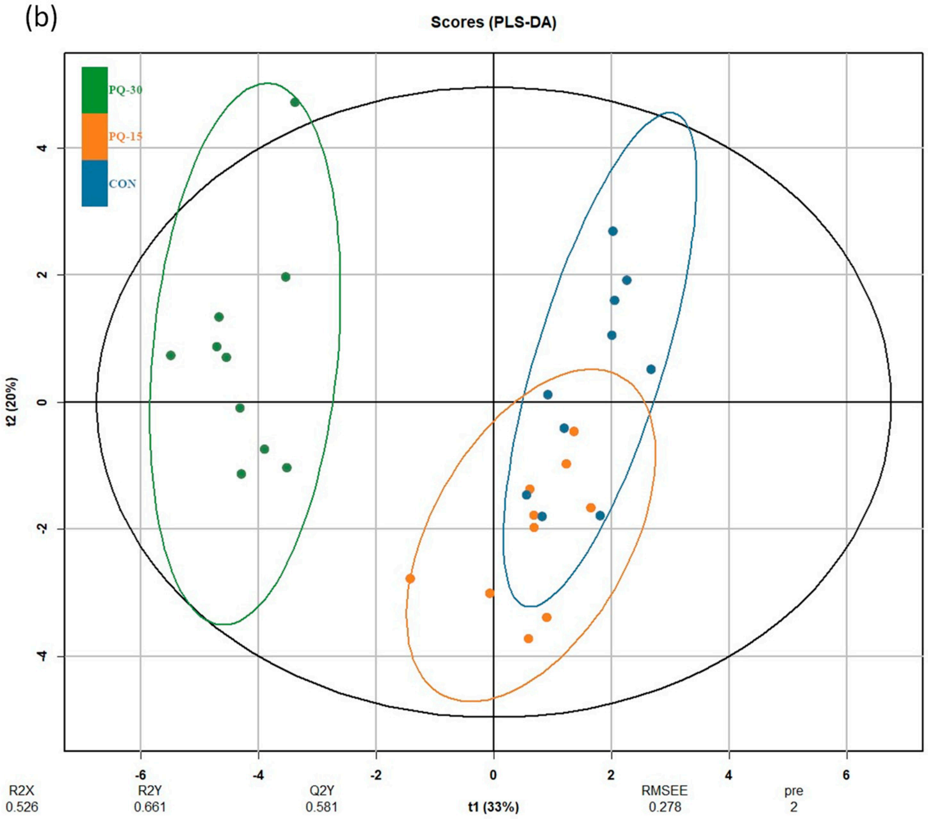

Supplementary Figure S5. Multivariate analysis's summary of urine metabolome in the PQ-induced oxidative stress model. (a) PCA scores of urine metabolome. (b) PLS-DA scores of urine metabolome. The Score plot is represented, and it includes the number of components, the cumulative R2X, R2Y and Q2Y. Groups (*n* = 10 animals per group): CON, blue; PQ-15, orange; PQ-30, green.

Supplementary Table S4. Summary of bacteria phyla in the CON, PQ-15 and PQ-30 groups (*n* = 8 animals per group). The summary of univariant analysis is shown including results of Kruskal-Wallis (p-value), Kruskal-Wallis corrected by HS (q-value) and FC, the statistically significant values (< 0.05) are highlighted in bold. Taxonomic data is presented as the mean of relative abundance (%).

	CON vs PQ-15 vs PQ-30		PQ-15 vs PQ-30			CON vs PQ-30			CON vs PQ-15			Relative abundance (%)		
Phylum	*p*-value	*q*-value	*p*-value	*q*-value	FC	*p*-value	*q*-value	FC	*p*-value	*q*-value	FC	CON	PQ-15	PQ-30
Bacteroidetes	**<0.01**	**<0.01**	**<0.01**	**<0.01**	0.02	**<0.01**	**<0.01**	0.02	0.90	0.95	1.20	58%	70%	1%
Firmicutes	**<0.01**	**<0.01**	**<0.01**	**0.01**	0.15	**<0.01**	**<0.01**	0.10	0.14	0.53	0.70	27%	19%	3%
Proteobacteria	**<0.01**	**0.01**	**0.01**	**0.01**	12.48	**<0.01**	**0.02**	7.05	0.76	0.95	0.56	11%	6%	76%
Verrucomicrobia	**<0.01**	**<0.01**	**<0.01**	**0.01**	7.02	**<0.01**	**<0.01**	8.38	0.62	0.95	1.19	2%	3%	19%
Deferribacteres	**0.02**	**0.02**	**0.03**	**0.01**	0.20	**0.01**	**0.05**	0.33	0.22	0.63	1.60	1%	2%	0.3%
Actinobacteria	**0.01**	**0.01**	**0.02**	**0.01**	-	**0.01**	**0.05**	2.16	0.06	0.30	-	0.1%	0.0%	0.2%

Supplementary Table S5. Summary of bacteria species in the CON, PQ-15 and PQ-30 groups (*n* = 8 animals per group). The summary of univariant analysis is shown including results of Kruskal-Wallis (*p*-value), Kruskal-Wallis corrected by Holm-Šídák (*q*-value) and FC, the statistically significant values (< 0.05) are highlighted in bold. Taxonomic data is presented as the mean of relative abundance (%).

	CON vs PQ-15 vs PQ-30		PQ-15 vs PQ-30			CON vs PQ-30			CON vs PQ-15			Relative abundance (%)		
Specie	*p*-value	*q*-value	*p*-value	*q*-value	FC	*p*-value	*q*-value	FC	*p*-value	*q*-value	FC	CON	PQ-15	PQ-30
Muribaculaceae bacterium DSM 103720	**<0.01**	**<0.01**	**<0.01**	**0.02**	-	**<0.01**	**<0.01**	0.00	0.46	1.00	1.13	30.40%	34.43%	0.03%
Muribaculum intestinale	**<0.01**	**<0.01**	**<0.01**	**0.02**	-	**<0.01**	**<0.01**	0.00	0.71	1.00	1.00	18.49%	18.51%	-
Escherichia coli	**<0.01**	**0.01**	**<0.01**	**0.02**	12.48	**<0.01**	**<0.01**	7.05	0.81	1.00	0.56	10.79%	6.09%	76.00%
Lachnospiraceae bacterium 28 4	0.35	0.82	0.17	0.66	0.05	0.50	0.97	0.04	0.30	1.00	0.81	8.60%	6.94%	0.32%
Lachnospiraceae bacterium 10 1	**0.01**	0.10	0.34	0.66	0.01	**<0.01**	**0.02**	0.01	0.16	0.98	0.69	6.44%	4.46%	0.06%
Lactobacillus murinus	**0.02**	0.17	0.14	0.66	1.28	0.20	0.83	0.18	**0.01**	0.19	0.14	5.88%	0.84%	1.07%
Parabacteroides goldsteinii	**0.03**	0.22	**0.02**	0.24	0.08	0.27	0.89	0.24	**0.04**	0.71	3.15	4.80%	15.13%	1.15%
Bacteroides uniformis	**<0.01**	**0.03**	**0.04**	0.28	0.04	**<0.01**	**0.01**	0.02	0.10	0.92	0.48	4.55%	2.18%	0.10%
Akkermansia muciniphila	**<0.01**	**<0.01**	**<0.01**	**0.02**	7.02	**<0.01**	**<0.01**	8.38	0.50	1.00	1.19	2.30%	2.75%	19.31%

Oscillibacter sp 1 3	**<0.01**	**0.01**	**<0.01**	**0.02**	0.02	**0.01**	0.08	0.05	**0.03**	0.55	2.00	1.90%	3.80%	0.09%
Anaerotruncus sp G3 2012	**<0.01**	**0.01**	**<0.01**	**0.02**	0.03	**<0.01**	**<0.01**	0.02	0.85	1.00	0.77	1.87%	1.44%	0.04%
Lachnospiraceae bacterium COE1	0.35	0.82	0.14	0.66	-	0.62	0.97	0.00	0.30	1.00	0.69	1.63%	1.12%	0.00%
Mucispirillum schaedleri	0.08	0.43	0.05	0.31	0.20	0.27	0.89	0.33	0.09	0.90	1.60	1.00%	1.60%	0.33%
Lactobacillus taiwanensis	**0.01**	0.12	**<0.01**	**0.02**	-	0.07	0.50	1.02	0.18	0.99	-	0.26%	-	0.27%
Ruthenibacterium lactatiformans	**<0.01**	**0.03**	**<0.01**	**0.02**	22.24	**<0.01**	**0.04**	2.99	0.58	1.00	0.13	0.23%	0.03%	0.69%
Firmicutes bacterium ASF500	**0.01**	0.14	**0.01**	0.11	0.10	0.06	0.48	0.18	0.08	0.88	1.87	0.21%	0.39%	0.04%
Lachnospiraceae bacterium A4	0.77	0.82	0.29	0.66	-	0.76	0.97	0.00	0.90	1.00	0.98	0.17%	0.17%	-
Bacteroides caecimuris	0.41	0.82	0.29	0.66	2.02	0.54	0.97	0.41	0.24	1.00	0.20	0.13%	0.03%	0.05%
Lactobacillus reuteri	0.24	0.74	0.14	0.66	0.39	0.13	0.70	0.26	1.00	1.00	0.67	0.11%	0.08%	0.03%
Enterorhabdus caecimuris	**0.01**	0.14	**0.01**	0.11	-	**0.01**	0.14	0.41	0.58	1.00	0.00	0.08%	-	0.03%
Lactobacillus johnsonii	**<0.01**	**0.02**	**<0.01**	**0.03**	31.23	**<0.01**	**0.01**	2.22	0.50	1.00	0.07	0.07%	0.01%	0.16%
Romboutsia ilealis	**0.02**	0.17	**0.03**	0.24	0.48	**0.01**	0.10	0.15	0.50	1.00	0.32	0.04%	0.01%	0.01%
Acutalibacter muris	0.12	0.55	**0.02**	0.24	-	0.67	0.97	0.00	0.20	0.99	-	0.04%	-	-
Bifidobacterium pseudolongum	**<0.01**	**0.01**	**<0.01**	**0.02**	-	**<0.01**	**<0.01**	-	0.54	1.00	-	-	-	0.15%
Enterococcus faecalis	**<0.01**	0.09	**0.02**	0.24	-	**<0.01**	**0.04**	-	0.54	1.00	-	-	-	0.01%
Blautia coccoides	**0.01**	0.10	**0.02**	0.24	-	**<0.01**	**0.04**	-	0.54	1.00	-	-	-	0.02%
Faecalibaculum rodentium	**<0.01**	**0.04**	**<0.01**	0.05	-	**<0.01**	**0.04**	-	0.54	1.00	-	-	-	0.04%

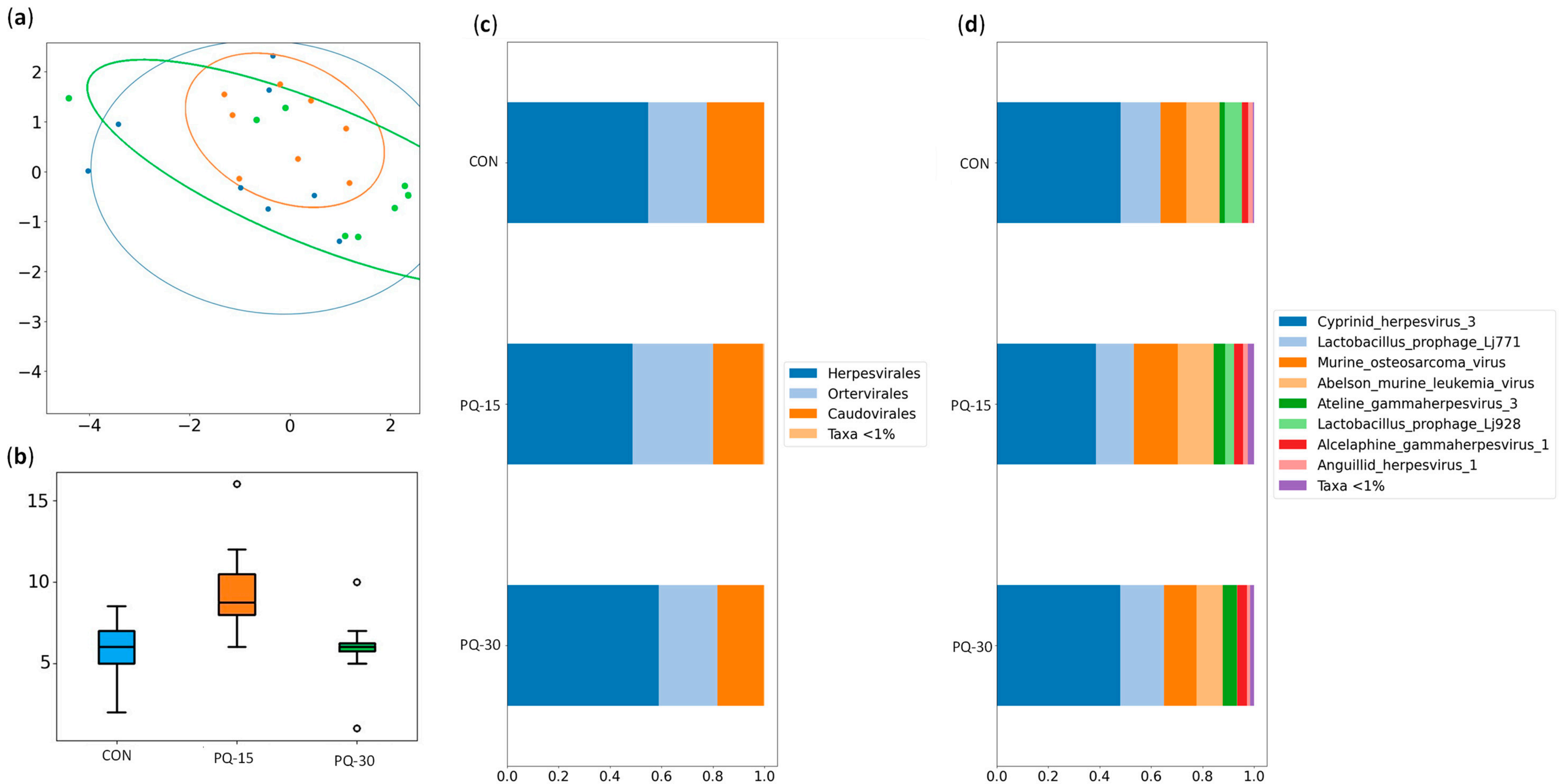

Supplementary Figure S6. Summary of the virus statistical analysis in the PQ-induced oxidative stress model. (a) Beta diversity: PCA plot calculated by Aitchison distance. (b) Alpha diversity (AU): Chao1 index. (c) Relative distribution of virus phylum. (d) Relative distribution of virus species. Groups (n = 8 animals per group): CON, blue; PQ-15, orange; PQ-30, green.

Supplementary Table S6. Summary of virus species in the CON, PQ-15 and PQ-30 groups (*n* = 8 animals per group). The summary of univariant analysis is shown including results of Kruskal-Wallis (*p*-value), Kruskal-Wallis corrected by Holm-Šídák (*q*-value) and FC, the statistically significant values (< 0.05) are highlighted in bold. Taxonomic data is presented as the mean of relative abundance (%).

	CON vs PQ-15 vs PQ-30		PQ-15 vs PQ-30			CON vs PQ-30			CON vs PQ-15			Relative abundance (%)		
Specie	*p*-value	*q*-value	*p*-value	*q*-value	FC	*p*-value	*q*-value	FC	*p*-value	*q*-value	FC	CON	PQ-15	PQ-30
Cyprinid herpesvirus 3	**<0.01**	0.06	**0.02**	0.29	1.25	0.46	0.99	1.00	**<0.01**	**0.02**	0.80	48.08%	38.45%	47.96%
Lactobacillus prophage Lj771	0.25	0.90	0.17	0.78	1.15	0.13	0.93	1.10	0.85	0.97	0.95	15.51%	14.80%	16.99%
Abelson murine leukemia virus	0.79	0.96	1.00	1.00	0.73	0.81	0.99	0.80	0.43	0.97	1.10	12.82%	14.10%	10.23%
Murine osteosarcoma virus	0.78	0.96	0.40	0.88	0.74	0.95	0.99	1.25	0.62	0.97	1.68	10.12%	16.98%	12.61%
Lactobacillus prophage Lj928	0.07	0.53	**0.03**	0.34	0.04	0.08	0.82	0.02	0.39	0.97	0.53	6.68%	3.53%	0.16%
Alcelaphine gammaherpesvirus 1	0.57	0.96	0.29	0.88	1.08	0.43	0.99	1.59	0.76	0.97	1.48	2.41%	3.56%	3.83%
Ateline gammaherpesvirus 3	0.08	0.58	0.14	0.75	1.28	0.04	0.58	2.69	0.36	0.97	2.10	2.06%	4.32%	5.53%
Anguillid herpesvirus 1	0.55	0.96	0.60	0.88	0.63	0.39	0.99	0.61	0.39	0.97	0.97	1.91%	1.85%	1.17%
Pestivirus Giraffe 1	0.15	0.77	0.07	0.66	0.85	0.24	0.99	2.14	0.22	0.89	2.53	0.16%	0.40%	0.34%
Human alphaherpesvirus 2	**<0.01**	**0.02**	**0.01**	0.17	-	0.10	0.89	0.00	**<0.01**	**0.02**	-	0.14%	0.00%	0.00%
Bovine alphaherpesvirus 1	0.39	0.92	0.17	0.78	0.83	0.39	0.99	2.97	0.50	0.97	3.58	0.13%	0.45%	0.37%
Salmonella phage RE 2010	**0.04**	0.41	0.07	0.66	-	0.22	0.99	-	0.02	0.26	-	-	0.67%	-
Enterobacteria phage mEp460	**0.04**	0.41	0.07	0.66	-	0.22	0.99	-	0.02	0.26	-	-	0.22%	-
Enterococcus phage phiEf11	**<0.01**	**0.04**	0.01	0.14	-	0.71	0.99	-	**<0.01**	**0.02**	-	-	0.00%	0.81%
Feline leukemia virus	**0.04**	0.41	0.07	0.66	-	0.22	0.99	-	0.02	0.26	-	-	0.21%	-
Koala retrovirus	0.25	0.90	0.34	0.88	-	0.22	0.99	-	0.18	0.88	-	-	0.14%	-
Chrysodeixis chalcites nucleopolyhedrovirus	0.25	0.90	0.34	0.88	-	0.22	0.99	-	0.18	0.88	-	-	0.26%	-
Molluscum contagiosum virus	**0.04**	0.41	0.07	0.66	-	0.22	0.99	-	0.02	0.26	-	-	0.04%	-

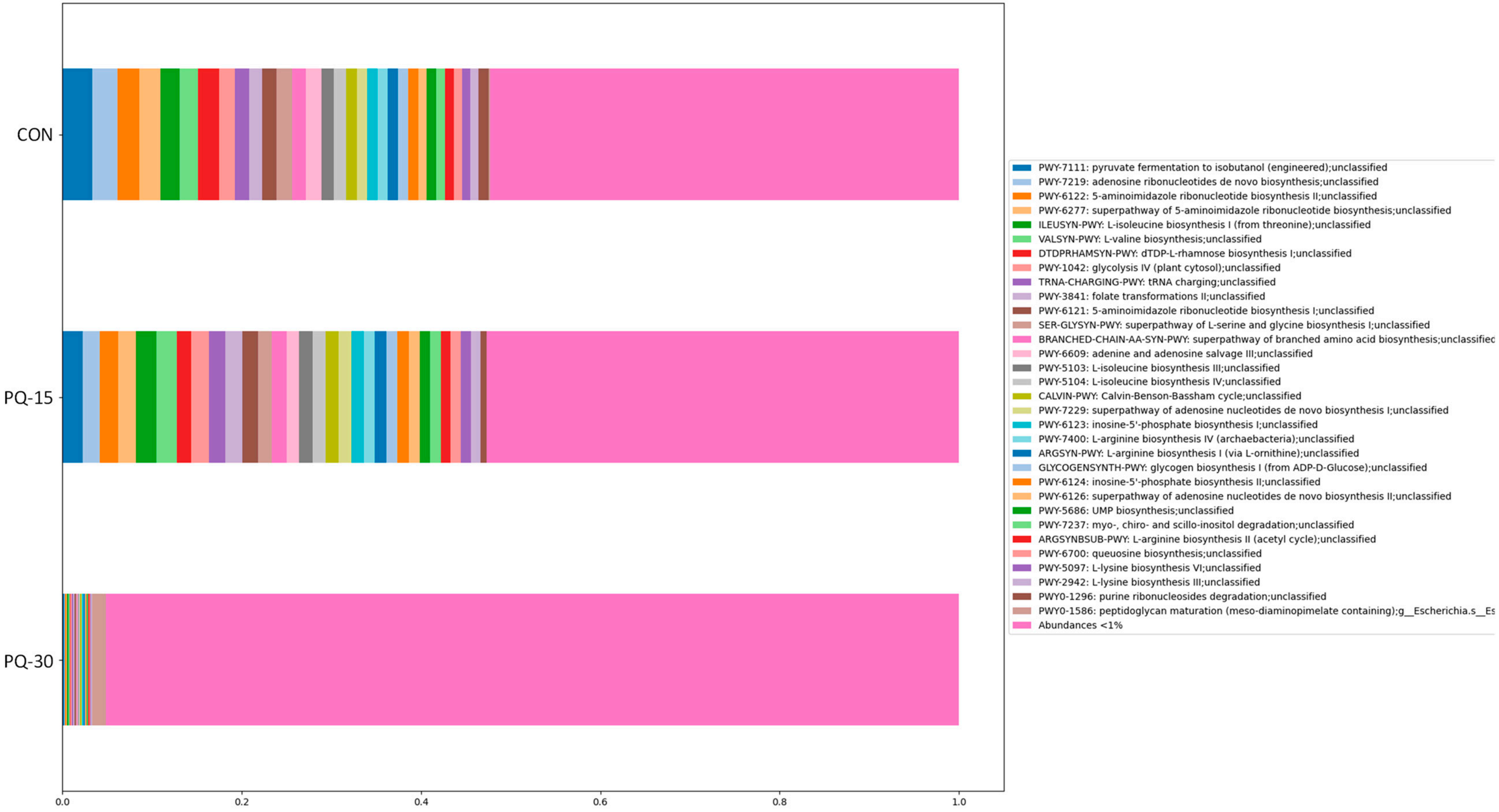

Supplementary Figure S7. Relative distribution of functions between the experimental groups (*n* = 8 animals per group) of the PQ-induced oxidative stress model.

Supplementary Table S7. Statistically significant differences in functions between the experimental groups of the PQ-induced oxidative stress model. In this table, the most abundant functions are represented in CON, PQ-15 and PQ-30 groups (*n* = 8 animals per group). The summary of analysis is shown including results of Kruskal-Wallis corrected by HS (*q*-value) and % relative of each function, the statistically significant values (< 0.05) are highlighted in bold.

	Corrected *p*-value				Relative %		
	CON vs PQ-15 vs PQ-30	CON vs PQ-15	PQ-15 vs OXS B	CON vs OXS B	CON	PQ-15	PQ-30
PWY-7111: pyruvate fermentation to isobutanol (engineered)	**0.001**	0.462	**0.001**	**0.001**	3.60%	2.44%	1.31%
PWY-7219: adenosine ribonucleotides de novo biosynthesis	**0.001**	0.854	**0.001**	**0.001**	2.95%	2.04%	0.65%
PWY-6122: 5-aminoimidazole ribonucleotide biosynthesis II	**0.001**	0.582	**0.001**	**0.001**	2.56%	2.15%	0.69%
PWY-6277: superpathway of 5-aminoimidazole ribonucleotide biosynthesis	**0.001**	0.582	**0.001**	**0.001**	2.56%	2.15%	0.69%
DTDPRHAMSYN-PWY: dTDP-L-rhamnose biosynthesis I	**0.001**	0.540	**0.001**	**0.001**	2.43%	1.65%	0.14%
VALSYN-PWY: L-valine biosynthesis	**0.004**	0.854	**0.001**	**0.004**	2.30%	2.45%	1.21%
ILEUSYN-PWY: L-isoleucine biosynthesis I (from threonine)	**0.003**	0.582	**0.001**	**0.003**	2.21%	2.32%	1.21%
PWY-6609: adenine and adenosine salvage III	**<0.001**	0.270	**0.001**	**0.000**	1.86%	1.50%	0.41%
PWY-1042: glycolysis IV (plant cytosol)	**0.002**	0.501	**0.001**	**0.003**	1.81%	2.02%	0.67%
SER-GLYSYN-PWY: superpathway of L-serine and glycine biosynthesis I	**0.000**	0.245	**0.001**	**0.001**	1.80%	1.52%	0.48%
TRNA-CHARGING-PWY: tRNA charging	**0.002**	0.358	**0.001**	**0.004**	1.70%	1.98%	0.55%
PWY-6121: 5-aminoimidazole ribonucleotide biosynthesis I	**0.002**	0.391	**0.001**	**0.003**	1.67%	1.87%	0.64%
BRANCHED-CHAIN-AA-SYN-PWY: superpathway of branched amino acid biosynthesis	**0.003**	0.713	**0.001**	**0.003**	1.64%	1.68%	0.94%
PWY-3841: folate transformations II	**0.001**	0.111	**0.001**	**0.004**	1.57%	1.92%	0.40%
PWY-5103: L-isoleucine biosynthesis III	**0.003**	0.668	**0.001**	**0.003**	1.52%	1.54%	0.90%
PWY-5104: L-isoleucine biosynthesis IV	**0.004**	0.713	**0.001**	**0.004**	1.37%	1.46%	0.51%
CALVIN-PWY: Calvin-Benson-Bassham cycle	**0.002**	0.854	**0.001**	**0.002**	1.33%	1.44%	0.67%
PWY-6123: inosine-5'-phosphate biosynthesis I	**0.003**	0.759	**0.001**	**0.004**	1.25%	1.43%	0.45%
PWY-7400: L-arginine biosynthesis IV (archaebacteria)	**0.002**	0.713	**0.001**	**0.003**	1.19%	1.29%	0.49%
ARGSYN-PWY: L-arginine biosynthesis I (via L-ornithine)	**0.002**	0.759	**0.001**	**0.003**	1.18%	1.28%	0.49%
GLYCOGENSYNTH-PWY: glycogen biosynthesis I (from ADP-D-Glucose)	**0.003**	0.759	**0.001**	**0.004**	1.18%	1.25%	0.42%
PWY-6124: inosine-5'-phosphate biosynthesis II	**0.003**	0.624	**0.001**	**0.003**	1.17%	1.34%	0.44%
PWY-7229: superpathway of adenosine nucleotides de novo biosynthesis I	**0.002**	0.178	**0.001**	**0.005**	1.17%	1.46%	0.51%
PWY0-1296: purine ribonucleosides degradation	**0.001**	0.426	**0.015**	**0.000**	1.17%	0.68%	0.40%
PWY-5686: UMP biosynthesis	**0.003**	0.854	**0.001**	**0.004**	1.14%	1.27%	0.52%

ARGSYNBSUB-PWY: L-arginine biosynthesis II (acetyl cycle)	**0.004**	0.713	**0.001**	**0.006**	1.07%	1.14%	0.63%
PWY-6700: queuosine biosynthesis	**0.002**	0.327	**0.001**	**0.004**	1.04%	1.28%	0.45%
PWY-6126: superpathway of adenosine nucleotides de novo biosynthesis II	**0.005**	0.221	**0.001**	**0.016**	1.01%	1.27%	0.45%

Suplementary Table S8. Multi-omics data association with isoprostanes (gold standard biomarker). The multi-omics data was given as input to MaAslin2 comprehensive R package (Multivariate Association with Linear Models 2, Version 1.8.0—Bioconductor) alongside isoprostanes levels. Results were considered significant if they had a q-value smaller than 0.25.

Feature type	Feature	Coef	stderr	*p*-value	q-value
Plasma metabolite	Glutamic.Acid	-0.18	0.02	<0.01	<0.01
Plasma metabolite	Hydroxyproline	-0.19	0.03	<0.01	<0.01
Plasma metabolite	Citric.Acid	-0.07	0.01	<0.01	<0.01
Plasma metabolite	LPC.18.2	-0.12	0.02	<0.01	<0.01
Plasma metabolite	LPC.20.0	-0.14	0.02	<0.01	<0.01
Plasma metabolite	PC.38.2	-0.10	0.02	<0.01	<0.01
Plasma metabolite	LPC.15.0	-0.11	0.02	<0.01	<0.01
Plasma metabolite	Serine	-0.18	0.04	<0.01	<0.01
Plasma metabolite	Ornithine	-0.15	0.03	<0.01	<0.01
Plasma metabolite	SM.36.1	0.19	0.04	<0.01	<0.01
Plasma metabolite	SM.36.2	0.14	0.03	<0.01	<0.01
Plasma metabolite	X3.Hydroxybutiric.Acid	0.14	0.03	<0.01	<0.01
Plasma metabolite	Proline	-0.12	0.03	<0.01	<0.01
Plasma metabolite	Aspartic.Acid	-0.08	0.02	<0.01	<0.01
Plasma metabolite	Valine	0.10	0.02	<0.01	<0.01
Plasma metabolite	Isoleucine	0.15	0.03	<0.01	<0.01
Plasma metabolite	LPC.18.1	-0.12	0.03	<0.01	<0.01
Plasma metabolite	Aconitic.Acid	-0.22	0.05	<0.01	<0.01
Plasma metabolite	PC.35.2	-0.07	0.02	<0.01	<0.01
Plasma metabolite	SM.38.1	0.13	0.03	<0.01	<0.01
Plasma metabolite	Glutamine	-0.28	0.07	<0.01	<0.01
Plasma metabolite	ChoE..22.6.	0.11	0.03	<0.01	0.01
Plasma metabolite	Cholesterol	-0.05	0.01	<0.01	0.01
Plasma metabolite	Phenylalanine	0.06	0.02	<0.01	0.01
Plasma metabolite	ChoE..18.2.	-0.05	0.01	<0.01	0.01
Plasma metabolite	Leucine	0.09	0.02	<0.01	0.01
Plasma metabolite	ChoE..18.3.	-0.10	0.03	<0.01	0.01
Plasma metabolite	ChoE..20.2.	-0.10	0.03	<0.01	0.01
‘Plasma metabolite	Glucose.6.Phosphate	-0.10	0.03	<0.01	0.01
Plasma metabolite	PC.40.4	0.09	0.02	<0.01	0.01
Plasma metabolite	PC.32.2	-0.14	0.04	<0.01	0.01
Plasma metabolite	SM.34.1	0.08	0.03	<0.01	0.01
Plasma metabolite	SM.34.2	0.06	0.02	<0.01	0.01
Plasma metabolite	Threonine	-0.04	0.01	<0.01	0.02
Plasma metabolite	Methionine	-0.05	0.02	<0.01	0.02
Plasma metabolite	LPC.16.0	-0.03	0.01	<0.01	0.02
Plasma metabolite	SM.35.1	0.10	0.03	<0.01	0.02
Plasma metabolite	Glyceric.Acid	-0.09	0.03	<0.01	0.02
Plasma metabolite	ChoE..16.1.	-0.16	0.05	0.01	0.02
Plasma metabolite	Glycerol	-0.05	0.02	0.01	0.02
Plasma metabolite	Fumaric.Acid	0.06	0.02	0.01	0.02

Plasma metabolite	Urea	0.04	0.01	0.01	0.02
Plasma metabolite	Alanine	-0.05	0.02	0.01	0.03
Plasma metabolite	Glycolic.Acid	0.05	0.02	0.01	0.03
Plasma metabolite	SM.39.1	0.08	0.03	0.01	0.03
Plasma metabolite	SM.42.3	0.08	0.03	0.01	0.03
Plasma metabolite	X2.Hydroxyglutaric	0.08	0.03	0.01	0.03
Plasma metabolite	ChoE..18.1.	-0.05	0.02	0.01	0.04
Plasma metabolite	PC.40.5	0.05	0.02	0.01	0.04
Plasma metabolite	PC.38.4	0.04	0.02	0.02	0.05
Plasma metabolite	LPC.18.0.e	-0.04	0.02	0.02	0.05
Plasma metabolite	SM.40.2	0.05	0.02	0.02	0.06
Plasma metabolite	Tyrosine	-0.08	0.03	0.03	0.06
Plasma metabolite	PC.33.1	-0.08	0.04	0.03	0.07
Plasma metabolite	Histidine	-0.10	0.05	0.04	0.09
Plasma metabolite	DG.34.3	-0.08	0.04	0.05	0.12
Plasma metabolite	PC.38.3	-0.08	0.04	0.06	0.13
Plasma metabolite	DG.36.4	-0.02	0.01	0.06	0.13
Plasma metabolite	Fructose.6.Phosphate	-0.06	0.03	0.07	0.14
Plasma metabolite	Asparagine	-0.07	0.03	0.07	0.15
Plasma metabolite	SM.33.1	0.03	0.02	0.08	0.17
Plasma metabolite	Beta.Alanine	-0.04	0.02	0.09	0.18
Plasma metabolite	DG.36.2	-0.04	0.02	0.09	0.18
Plasma metabolite	TG.50.4	-0.17	0.10	0.09	0.19
Plasma metabolite	PC.32.0	0.02	0.01	0.10	0.19
Plasma metabolite	Tryptophan	-0.05	0.03	0.11	0.21
Plasma metabolite	PC.32.1	-0.10	0.06	0.12	0.22
Urine metabolite	Fumarate	-0.47	0.09	<0.01	<0.01
Urine metabolite	Trigonelline	-0.38	0.07	<0.01	<0.01
Urine metabolite	Succinate	-0.18	0.04	<0.01	<0.01
Urine metabolite	Hippurate	-0.18	0.04	<0.01	<0.01
Urine metabolite	Citrate	-0.48	0.11	<0.01	<0.01
Urine metabolite	Tryptophan	-0.10	0.03	<0.01	<0.01
Urine metabolite	Methylamine	-0.11	0.03	<0.01	0.01
Urine metabolite	Glucose	-0.17	0.05	<0.01	0.01
Urine metabolite	Glycine	0.05	0.02	0.03	0.08
Urine metabolite	Dimethylsulfone	-0.05	0.02	0.04	0.08
Urine metabolite	X1.Mehtylnicotinamide	0.15	0.10	0.12	0.23
Urine metabolite	N.N.Dimethylglycine	0.08	0.05	0.14	0.24
Microorganism	Bifidobacterium.pseudolongum	0.23	0.03	<0.01	<0.01
Microorganism	Muribaculaceae.bacterium.DSM.103720	-1.09	0.23	<0.01	<0.01
Microorganism	Lactobacillus.johnsonii	0.30	0.07	<0.01	<0.01
Microorganism	Muribaculum.intestinale	-0.35	0.09	<0.01	<0.01
Microorganism	Akkermansia.muciniphila	0.72	0.18	<0.01	<0.01
Microorganism	Lactobacillus.prophage.Lj771	-0.30	0.08	<0.01	0.01
Microorganism	Escherichia.coli	0.35	0.12	0.01	0.04
Microorganism	Anaerotruncus.sp.G3.2012	-0.50	0.18	0.01	0.05

Microorganism	Lactobacillus.taiwanensis	0.80	0.32	0.02	0.06
Microorganism	Murine.osteosarcoma.virus	-0.29	0.12	0.02	0.07
Microorganism	Mucispirillum.schaedleri	-0.36	0.16	0.03	0.08
Microorganism	Lachnospiraceae.bacterium.10.1	-0.50	0.22	0.03	0.08
Microorganism	Bacteroides.uniformis	-0.40	0.20	0.06	0.15
Microorganism	Lactobacillus.prophage.Lj928	-0.17	0.09	0.08	0.17
Microorganism	Abelson.murine.leukemia.virus	-0.26	0.14	0.08	0.17
Microorganism	Lachnospiraceae.bacterium.COE1	-0.61	0.35	0.09	0.18

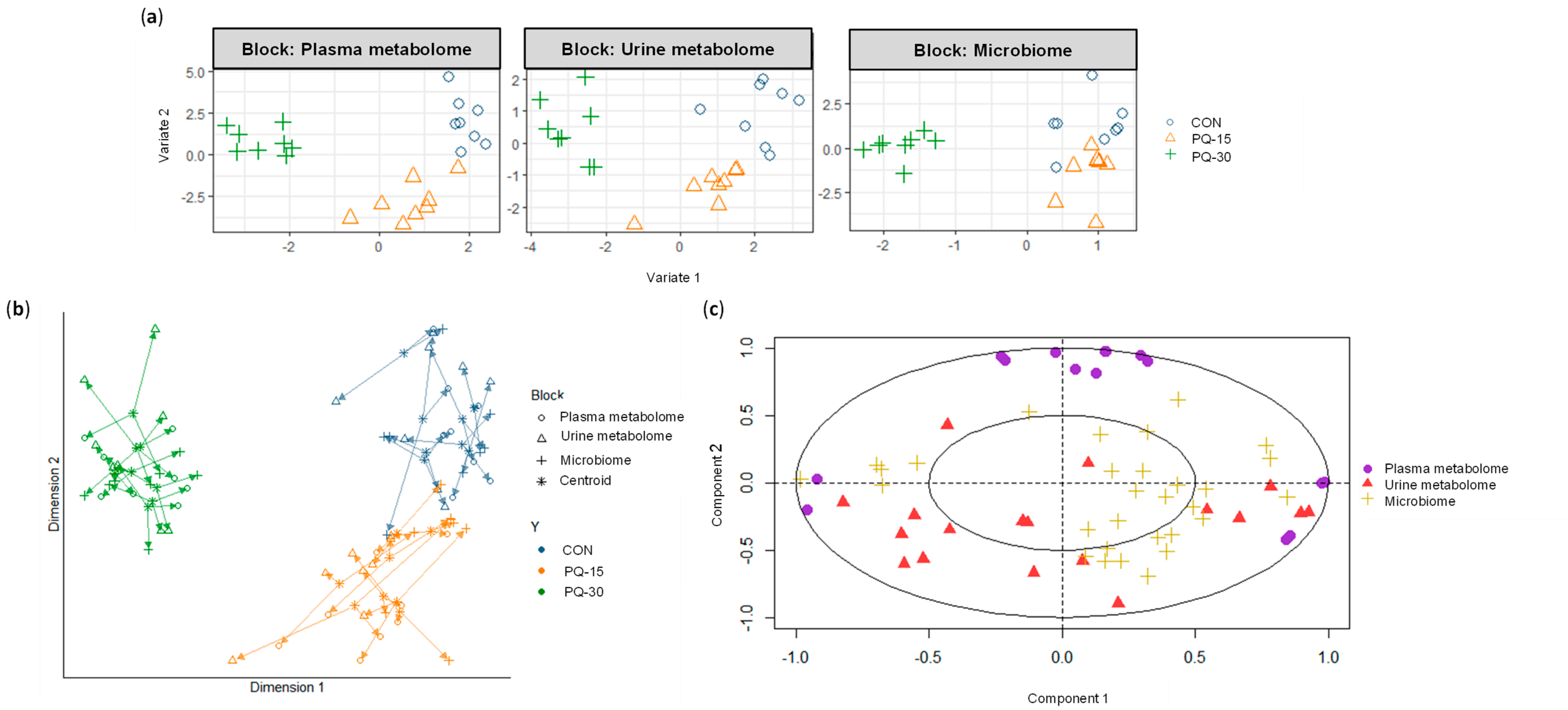

Supplementary Figure S8. Multi-omics data integration of plasma metabolome, urine metabolome and microbiome using DIABLO in the PQ-induced oxidative stress model. (a) Sample plot. The samples, which are plotted according to their scores on the 2 components for each data set, are associated showing the degree of agreement between the different data sets and the discriminative ability of each data set. Samples are coloured by group: CON; blue, PQ-15; orange and PQ-30; green. (b) Arrow plot. The samples are projected into the space spanned by the first 2 components for each data set then overlaid across data sets. The start of the arrow indicates the centroid between all data sets for a given sample and the tip of the arrow the location of the same sample in each block. Arrows further from their centroid indicate some disagreement between data sets. Samples are coloured by group (CON; blue, PQ-15; orange and PQ-3; green) and data sets are shaped (plasma metabolome: circle, urine metabolome: triangle and microbiome: cross). (c) Correlation circle plot. The plot highlights the potential associations within and between different variable types. Clusters of points indicate a strong

elation between variables. Each colour and shape indicate the type of features: plasma metabolome (purple circle), urine metabolites (red triangle) and finally, microbiome (yellow cross).

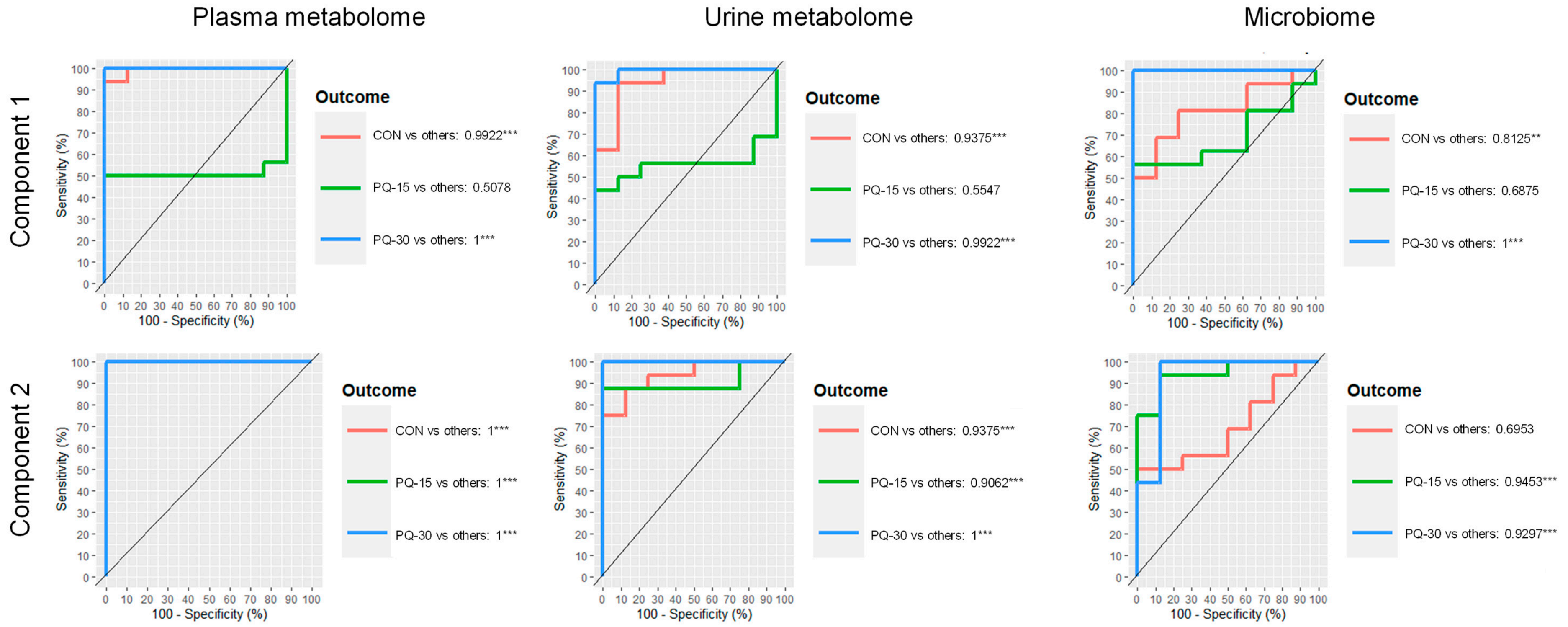

Supplementary Figure S9. ROC and AUC based on DIABLO performed on the PQ-induced oxidative stress model. This figure shows the ROC curve and AUC for one class *versus* the others for each data set (plasma metabolome, urine metabolome and microbiome) and the 2 components. The Wilcoxon test p-value is calculated to assess the differences between the predicted components from one class *versus* the others. ** $p < 0.05$ (significantly different) and *** $p < 0.01$ (high significantly different).
